# Supplementary material for: The Global Jukebox: A public database of performing arts and culture
Source: PLoS One. 2022 Nov 2;17(11):e0275469. doi: 10.1371/journal.pone.0275469 (PMC9629617; doi:10.1371/journal.pone.0275469)
Supplement: S1 Checklist — (PDF) [file pone.0275469.s001.pdf]

Inclusivity in global research

PLOS' policy on inclusivity in global research aims to improve transparency in the reporting of research performed outside of researchers' own country or community and ensures that PLOS publications reporting global research adhere to high standards for research ethics and authorship. Authors of relevant research articles may be asked to complete the questionnaire below, which outlines ethical, cultural, and scientific considerations specific to inclusivity in global research. This questionnaire may be requested when researchers have travelled to a different country to conduct research, if research uses samples collected in another country, research with Indigenous populations or their lands, or if research is on cultural artefacts. Researchers travelling to another country solely to use laboratory equipment will not normally be required to complete the questionnaire. However, the questionnaire can be requested at the journal's discretion for any submission – if you have been requested to complete this questionnaire by the PLOS journal you submitted to, please do so.

Please complete the questionnaire below and include this as a Supporting Information file with your manuscript. Note that if your paper is accepted for publication, this checklist will be published with your article in the supporting information files. Please ensure that you reference the checklist in the main body of your manuscript. We suggest adding a subsection 'Inclusivity in global research' to your Methods section and adding the following sentence: "Additional information regarding the ethical, cultural, and scientific considerations specific to inclusivity in global research is included in the Supporting Information (SX Checklist)"

The questions have been designed to be applicable to a wide range of study types, and there are subsections for both human subjects research and non-human subjects research. If any of the questions are not relevant to your research please mark them as "N/A" as appropriate.

Ethical considerations, permits and authorship

This section is applicable to all research types.

Provide details as to who granted permissions and/or consent for the study to take place in the Methods section of your manuscript. This should include the names of **all** ethics boards, governmental organizations, community leaders or other bodies that provided approval for the study. If individuals provided approval refer to these people by their role or title but do not list their name(s).

Reported on page number: The "Acknowledgments" section (pp. 31-32) points the reader to Lomax 1968:xv-xvii containing the full list of individuals and groups involved in the original collection of audio recordings and Cantometric data. The present study did not require new ethical approvals as it involved digitization, cleaning, validation, and reanalysis of previously published audio recordings and analyses, as described in the Introduction (pp. 5-9)).

If there were any deviations from the study protocol after approval was obtained please provide details of these changes in the Methods section of your manuscript.

Reported on page number: N/A (see above)

Did this study involve local collaborators that are residents of the country where the research was conducted or members of the community studied? If you do not have any authors from said communities, please provide an explanation for this below.

The "Acknowledgments" section points the reader to Lomax 1968:xv-xvii and the metadata at http://theglobaljukebox.org with detailed credits for each song containing the full list of individuals and groups involved in the original collection and publication of audio recordings. Like the *Ethnographic Atlas* that inspired it (digitized and republished by Kirby et al., 2016, *PLOS ONE*), the Global Jukebox data were first published before the standardization of ethical approval and reporting practices in the 1979 Belmont Report. The tradition in ethnomusicology at the time (and often still today) was generally to publish research results as sole-authored books, with contributions by musicians, other fieldworkers and assistants acknowledged but without coauthorship. At the time, Lomax's inclusion of formally coauthored chapters in his book was unusual, though today would fall short of the coauthorship expectations for cross-cultural research. Current standards of coauthorship on secondary analysis/reanalysis of previously published data are evolving, and it would have been impossible to include coauthors representing over 1,000 communities in the Global Jukebox, but our team of coauthors is affiliated with institutions in seven countries (USA, Japan, Italy, Germany, Canada, Australia, South Korea) and includes coauthors with roots in an additional seven countries (Uruguay, Venezuela, Morocco, Turkmenistan, New Zealand, Ireland, India).

Although procedures for obtaining informed consent had not been decisively codified before 1979 and the ideals of collaborative research and inclusion had not yet been formally incorporated into the practice of ethical research, the goals, values and procedures of the Global Jukebox research project--and of its research collaborators and contributors and of Lomax himself--met and even anticipated the principles of respect for persons, beneficence, and justice that are at the core of the Belmont Report in the following respects:

- From the outset, Lomax's intent was to share the research and its insights with the world at large, and especially with those people whose cultures were represented as well as the many related societies whose oral traditions and expressive systems are systemically undervalued, exploited and eroded. Lomax's goal was to make them known, appreciated and understood in a global framework. He reached out through popular media and publications, multicultural secondary education, advocacy, mentoring, and created tools for learning how to code and find patterns in music and dance. The Global Jukebox was intended to reconnect people from all backgrounds with their histories and heritage directly through the medium of their expressive traditions, and to provide a set of tools for learning about their own aesthetic values.
- During the initial data collection, Alan Lomax, his collaborators and recording contributors spent extensive time doing fieldwork in communities around the world making recordings, understanding the local context, developing new methodologies that could compare their music in more meaningful ways than could be captured by traditional Western staff notation (see Section 2.3."Coded

- To gauge the validity of the developing ideas, aims and methods of Cantometrics, Lomax consistently shared them with contributors, colleagues and collaborators from the field of many different backgrounds (e.g., the Rev. Frederick Douglass Kirpatrick, Worth Long, Bessie Jones, Jacob D. Elder, Mabel Hillery, Vera Ward Hall, Jorge Preloran, John Marshall, Allison Jablonko, Bill Broonzy, Subhendu Gosh, Jaap Kunst, Gilbert Rouget, Diego Carpitella, Winston Fleary, Zora Neale Hurston, John Marshall, New York City high school students, Seamus Ennis, Margaret Barry, Ewan McColl, John Henry Falk, Stetson Kennedy, Hamish Henderson, Jose Torner, Julio Caro Baroja).
- Lomax obtained permission from his interlocutors in the field to make recordings, publish them on records and in books, and research, consistently remunerated them--a rare and somewhat frowned upon practice at the time-- shared any forthcoming royalties, and protected recordings from appropriation and exploitation. These agreements are stored at the Association for Cultural Equity and the Library of Congress.
- Hundreds of contributing researchers obtained permissions following the ethical standards of their time; copies of such agreements are for the most part unavailable.

Current practices

- Our Terms and Conditions are modeled after Smithsonian Folkways Recordings, with which the Association for Cultural Equity (ACE) has a partnership.
- Our team of coauthors is affiliated with institutions in seven countries (USA, Japan, Italy, Germany, Canada, Australia, South Korea) and includes coauthors with roots in an additional seven countries (Uruguay, Venezuela, Morocco, Turkmenistan, New Zealand, Ireland, India).
- Contributors are acknowledged by name in the Acknowledgments Section of the Global Jukebox website (https://theglobaljukebox.org). Acknowledgments/credits of performers, fieldworkers, record labels, archives and repositories are included as metadata for each song at the GJB website.
- Rich metadata for each song and each society provides context, validity, and connections to other portals of knowledge and cross cultural data.
- The Jukebox focuses on women, children, and marginalized groups and minorities and more content of this kind is being developed in the form of Journeys and educational materials.

We engage in several types of community and international outreach with and through the Global Jukebox:

- Through ACE's work as an advisory NGO to UNESCO, we plan to collaborate internationally to obtain more recordings to balance out the sample and create curricula and Journeys on the Global Jukebox.
- Lomax’s recordings, films, photos, and notes have been repatriated to more than 80 localities, individuals, national and local libraries and education institutions, in four world regions, with more to come.
- Collaborative Journeys and Exhibits by artists and cultural leaders.
- The Global Jukebox includes a strong educational component which is being introduced into the Seattle, Washington and New York City school systems, and is used independently by many teachers.

- Our main objective is to engage communities and artists in creating content for the GJB, and together to make this a useful and meaningful resource for them and their children.
- We have made a commitment not to stream samples contributed by North American and Australian Indigenous peoples without their express permission.
- Displayed with each song, and elsewhere on the Global Jukebox, is an invitation for culture bearers, researchers, and the public to add corrections and make comments. There is a take down notice in the About section of the Global Jukebox.

For additional details about consultation with indigenous communities, repatriation efforts, and limiting availability of streaming audio files, section 6 ("Ethics, Rights and Consent").

Everyone listed as an author should meet PLOS' criteria for authorship and all individuals who meet these criteria should be included in the author byline, rather than the acknowledgements. Authorship criteria is based on the International Committee of Medical Journal Editors (ICMJE) Uniform Requirements for Manuscripts Submitted to Biomedical Journals - for further information please see here: https://journals.plos.org/plosone/s/authorship.

Human subjects research (e.g. health research, medical research, cross-cultural psychology)

Did you obtain written informed consent from a representative of the local community or region before the research took place? How did you establish who speaks for the community? Details of written informed consent obtained from study participants should be reported separately in the Methods section of your manuscript.

N/A (see above)

How did members of the local community provide input on the aims of the research investigation, its methodology, and its anticipated outcome(s)?

During the initial data collection, Alan Lomax, his collaborators and recording contributors spent extensive time doing fieldwork in communities around the world making recordings, understanding the local context, developing new methodologies that could compare their music in more meaningful ways than could be captured by traditional Western staff notation (see Section 1.3."Coded Performance Variables: Selection and Reliability" for details and references to debate about the pros and cons of this method).

When engaging with the local community, how did you ensure that the informed consent documents and other materials could be understood by local stakeholders?

N/A (see above)

Will the findings of the research be made available in an understandable format to stakeholders in the community where the study was conducted (e.g. via a presentation, summary report, copies of publications, etc.)? Please provide details of how this will be achieved.

The results are available in understandable formats at the the companion website, http://theglobaljukebox.org (e.g., "Education" and "Journeys" sections), as described in section S10 ("The Global Jukebox and Cultural Equity").

Non-human subjects research using specimens/ animals collected as part of the study, or those housed in archival collections. Examples include archaeology, paleontology, botany and zoology.

Did the permission you obtained from a local authority to perform the study include an agreement on access to outputs and benefit sharing? This may include procedures to enable fair distribution of the benefits and resources arising from the research performed. Please include any details of Prior Informed Consent and Benefit Sharing Agreements obtained. These may be required by field-specific regulations, for example the Convention on Biological Diversity (CBD) and the associated Nagoya Protocol.

N/A

If the material used in your study was imported, please A) provide the year it was imported and B) indicate whether permits were obtained to import/export the materials used, C) provide details of any permits obtained. If this information is not available, please indicate this.

N/A

If you used archival specimens, please state how the material used in your study was acquired by the institute it is held in and provide details of any permits obtained for the original excavations/ sample collection. If this information is not available, please indicate this.

N/A

How was the potential cultural significance of the materials collected in your study to local communities considered in your research design? Were Indigenous peoples and/or local researchers and institutions involved with archaeological excavations / collection of specimens? If so, please provide a description of their involvement.

N/A

If your manuscript includes photographs of human remains please indicate whether authors obtained permission from descendants or affiliated cultural communities to do so.

N/A
