# Supplementary material for: The Global Jukebox: A public database of performing arts and culture
Source: PLoS One. 2022 Nov 2;17(11):e0275469. doi: 10.1371/journal.pone.0275469 (PMC9629617; doi:10.1371/journal.pone.0275469)
Supplement: S1 File — This document includes all additional information and supplementary tables and figures referenced in the manuscript. This includes material regarding original data analysis and results, details of the Global Jukebox datasets, information on societies, data history, data normalization, coding reliability, data validation, current analyses, and the concept of cultural equity. (PDF) [file pone.0275469.s002.pdf]

S1. Supplementary material

S1.1. The Expressive Style and Culture Project

S1.1.1. Aesthetics and evolution

The cross-cultural investigation of expressive style made by Alan Lomax and his collaborators strongly suggests that aesthetic choices in music, dance, and other expressive arts are drawn from many possible combinations of performance features that reflect specific aspects of social organization and environment. They looked for the most conservative aspects of folk and indigenous performance: a hypothetical backbone of formal elements serving as a template for expressive style within cultures and regions of culture.

Lomax drew from biology, psychoanalysis, anthropology, ethology, and social history, but his chief sources were his interlocutors in the field, in the U.S, the Caribbean, Europe, former Soviet Union, and North Africa [1]. Performance and sociocultural data used in Lomax's research are the product of field research by the ethnographers (including by Murdock himself) whose work underpins the Ethnographic Atlas and Outline of World Cultures, and by ethnomusicologists and folklorists (including Lomax himself) whose recordings and observations contributed to the primary data.

Conrad M. Arensberg, Lomax's co-principal investigator, was an interactionist and cultural theorist who proposed a model of culture as emergent from minimal sequences of behavior within small groups [2]. Arensberg and Lomax were convinced by Raymond Birdwhistell's frame-by-frame analysis of film showing a constant stream of repeating nonverbal signals that synchronized individuals participating in a conversation [3]. They theorized that analogous sets of audible markers occur in singing within culturally acceptable ranges, making possible an infinite variety of choices within an overall design. Variations and departures from such ranges are signs of innovation, borrowing, or random or intentional change. They further conjectured that such templates are formative elements in the evolution of culture [4, 5], along with mechanisms of selection that are continually in play.

S1.1.2. The Contemporary View

While not universally welcomed, systematic comparative research is reentering the domains of the arts and humanities [6]. Lomax's sweeping pronouncements could class him as a "grand theorist." Nonetheless, he and his colleagues were engaged in a modern endeavor, using cross-cultural analysis in a series of experiments delving into questions which are admittedly broad but defined and delimited by strict protocols of analysis, data points, and probability. Recent studies deal with musical universals, perception and taste, cultural and musical evolution, and the role of aesthetics in society [7-9, 60]. To effectively investigate such questions, it is useful to supplement theory underpinning or arising from detailed studies of a single society or expressive phenomenon with approaches from other methodological vantage points, including comparative and cross cultural analysis. In contemporary archaeology there is a call for multiple approaches to research problems, ranging from world systems and historical perspectives to critical qualitative studies and comparative and quantitative analyses. [10]. Variation on the global scale isn't random; there are myriad global patterns that call for explanation. While each society is unique and interesting in its own right, it is also necessary to explain the broad cross-cultural patterns we see in culture and, in this case, music. [11-13].

S1.2. Original data analysis and results

Lomax (1980) summarized the main methods and findings from the Cantometrics Project, and this was further summarized and reviewed by Savage (2018) and Wood (2018a, 2018b; 2021). Here we provide a condensed summary--please see these other publications for further details.

S1.2.1. Original project: factor analysis of variables

Several discoveries emerged from Lomax's cross-cultural study of singing style. First, **factor analysis grouped the coding variables into nine sets** (plus four unique variables), revealing how the variables work together to define the elements of style (Table S1). New research grouping musical variables with like functions illustrates the strengths of this approach [14].

Table S1. **Cantometrics Factors** [15] (cf. Lomax 1968 Table 2 and Savage 2018 Fig. 2)

**Factor**	**Variables**		**Factor**	**Variables**
Factor 1: Differentiation Includes measures of articulation capacity or information potential	Precision of Enunciation (37)		Factor 5: Choral Organization	Musical Organization/Vocal Group (4)
	Interval Size (21)			Proportion of Solo to Group Performance (1)
	Repetition of Text (10)		Factor 6: Noise-Tension Level	Nasality (34)
Factor 2: Ornamentation	Glottal Ornament (31)			Rasp (35)
	Tremolo (30)			Vocal Width/Narrowness (33)
	Glissando (28)		Factor 7: Energy Level	Volume (25)
	Melisma (29)			Emphasis (Accent) (36)
	Embellishment (23)			Vocal Pitch (Register) (32)
Factor 3: Orchestral Organization	Musical Organization/Orchestra (7)		Factor 8: Rhythm	Overall Vocal Rhythm (11)
	Rhythmic Organization within the Orchestra (14)			Overall Orchestral Rhythm (13)
	Social Organization/Orchestra (3)			Tempo (24)
	Orchestral Blend (8)			Level of Melodic Variation (16)
	Rhythmic Relationship Voice to Orchestra (2)		Factor 9: Melody	Melodic Form (16)
Factor 4: Vocal Cohesiveness	Rhythmic Coordination/Vocal Group (6)			Number of Phrases (18)
	Rhythmic Relationship within the Vocal Group (12)			Phrase Symmetry (18)
	Social Organization/Vocal Group (1)		Unconnected Uniques	Phrase Length (17)

	Tonal Blend/Vocal Group (5)			Melodic Range (20)
				Position of Final Note (19)
				Polyphonic Type (22)

S1.2.2. Original project: clusters of musical style

Secondly, 10-14 broad but distinctive clusters of musical style emerged from factor analysis (Fig 1; Table S2). Although a new study finds that music is weakly related to genetic distance, language (basic vocabulary), and geographic proximity [14], the musical style clusters are roughly consistent with findings on human settlement made by geneticists and archeologists, and subgroupings within these regions matched their cultural geography; Kubik, for example, noted that Cantometrics's subregional clusters of African musical traditions agreed with his own findings [16].

Table S2. Cantometrics Song Style Cluster Descriptions [17, 18]

**1) African Gatherer:** Harmonious, inclusive, integrated music. "Group singing is not only contrapuntal but polyrhythmic, a playful weaving of four and more strands of short, flowing, canon-like melodies (each voice imitating the melody of the others), sounding wordless streams of vowels in clear, bell-like yodelling voices" (Lomax, 1976, p. 38).
**2) Proto-Melanesia:** "a low-energy, diffuse, harmonizing style" (Lomax, 1976, p. 40).
**3) Siberian:** "A guttural, raspy, punchy, slurred, nonsense-syllable kind of soloizing in extremely uneven phrases" (Lomax, 1976, p. 38).
**4) Circum-Pacific:** "North American indigenous songs of this family are often performed by men with notable vigor. Wide intervals and vocables are woven into freely structured, complex strophic forms consisting of several phrases repeated in a loose pattern. Complex, irregular vocal rhythms accompanied by one-beat time. The main choral style is often resonant, pulsing, and freely diffuse. Amongst Plains peoples, a narrow-voiced, high-pitched ululating vocal technique developed. In the settled clan villages of the southeastern and southwestern U.S. and in eastern Brazil another variant is a striking tonal and rhythmic blend with wider, deep voices" (Lomax, 1976, p. 38).
**5) Nuclear America:** "diffuse, highly individualized choralizing... use of polyphony that often veers toward vocal heterophony. Frequency of irregular and one-beat meters, wide melodic intervals, and guttural vocalizing link the Nuclear American style with that of the Sibero-American hunters—but a pattern of soft,

non raspy, high-pitched delivery points in another direction" (Lomax, 1976, p. 39).
**6) Tropical Gardeners:** "wide-voiced, superbly cohesive, polyphonic choralizing found among tropical village gardeners from Nigeria to the South Pacific island of Pukapuka" (Lomax, 1976, p. 41) Playful use of register, rasp, volume, emphasis, nasality, narrowing, yodeling, and other vocal effects as intermittent decorative motifs. Song leaders and instrumentalists constantly shift roles, vary the melody, complicate the rhythmic pattern, find new chords, increase tension and volume, then relax with accompanying voices and instruments in flowing synchrony. Overlapping of vocal parts, creating polyrhythmic interaction between voices and instruments, choruses and orchestras.
**7) Malayo-Polynesia:** "social unison with an unsurpassed cohesiveness, in spite of the fact that their complex society and their concern with genealogies motivate them to sing long, complexly textured poems in precise enunciation. Many of these chants are restrained within a narrow compass of notes, are dominated by free or irregular meters, and have a drone harmonic structure (i.e., a melody sung against a long, sustained note)" (Lomax, 1976, p. 40).
**8) Central Asia:** "in the bardic vein—where extremely virtuosic solo singers, employing superbly clear voices, accompanying themselves on various kind of lutes in free rhythm, perform miracles of ornamentation that add color to their great epics and elaborate lyrics" (Lomax, 1976, p. 42) One or two phrase melodies with wide intervals and performed in solo, often in a throaty voice. Irregular rhythms.
**9) Old High Culture:** "flowery textual style and...long, through-composed (non-strophic) melodies that are elaborately enunciated and heavily ornamented with passing notes, quavers, glottal shakes, melismata (many notes to one syllable), glides, and rhythmic variation...the vocal delivery tends to be tense, high-pitched, and nasal...solo bardic performances...Sometimes singing without accompaniment, more often accompanied by one heterophonically related instrument or by a large and elaborate orchestra..." (Lomax, 1976, p. 46).
**10) Western Europe:** "unaccompanied solo narrative song...The core of this tradition is compact strophes (stanzas) composed of 3–8 diatonic phrases of moderate length" (Lomax, 1976, pp. 44–45) Singers as neutral-voiced storytellers; refrains in hearty unison.

Regression and componential analysis of these clusters, along with productive, gender, and stratification factors derived from EA codes, implied evolutionary relationships between them. Refer to the Social Factors codebook and coding guide [19] for a detailed breakdown of how selected Ethnographic Atlas codes were applied to the societies sampled in the expressive style datasets and used for correlational analyses. From such experiments Lomax was able to outline socio-musical areas in considerable detail and in rough temporal sequences among and within, for example, Africa, Northern Amerindia, and Oceania [15]. Musical markers suggested close ties between distant societies in the remote past (for

example, between C. African and S. African hunter gatherers [20, 21], and between Tupian speaking Amazonian peoples and Melanesians), which were confirmed through ancient DNA analyses decades later [22, see also 21].

S1.2.3. Recent similar outcomes

Several unrelated studies have found seven to fourteen regions which grouped coded variable states together geographically [23-25]. They match Lomax's geographies of style, which for the most part match regions of (mostly) ancient settlement and migration found in genetic and archeological studies of old populations. A modern cluster analysis of 4,714 musical samples coded in Cantometrics using latent class analysis, found that, in tests modeling from one to 31 solutions, the 14-cluster solution and 2-3 of its nearest neighbors were the best "fit" to the data [26].

S1.2.4. Original correlation analyses

The societies included in the Ethnographic Atlas had been coded by Murdock for social and cultural characteristics documented by ethnographic research; these data have recently been validated [27]. The sociocultural variables were ethnographically documented between about 1650 and the 1960s (with a few much earlier exceptions).

Correlation analyses were run on every performance variable against many Ethnographic Atlas variables. This process helped identify the principal environmental and socio-cultural variables that varied with performance. A number of these were included in a set of 38 socio-cultural variables, some with modifications [19]. The societies in all the studies were then coded for these variables. These codings were tested against performance traits. Only statistically significant correlations ($p < .05$) were reported in publications. Questions about control for multiple comparisons and for autocorrelation were raised by Erickson (1976); but his reevaluation confirmed the cluster analysis and one of the earliest correlations in Cantometrics between pinched voice and nasality and restrictions on female sexuality [17, 28] finding that sexual restrictiveness predicts less sonority in singing.

This made it possible to intercorrelate musical and socioeconomic factors, and to distinguish two larger independent systems of interrelated musical factors labeled

“Differentiative" (Information-Productivity') and “Integrative” (Labor Organization-Gender Role) with factors of dynamics, tension, ornament, melody, rhythm in a dependent and variable relationship to both. Plotting the two large systems (Integrative and Differentiative) on a vertical axis (left to right) against a horizontal axis bearing the regional cultural systems (Fig 1; Table S2), shows how the Integrative system varies its trajectory in response to the Differentiative system, and how both correlate with cultural systems (Fig S1).

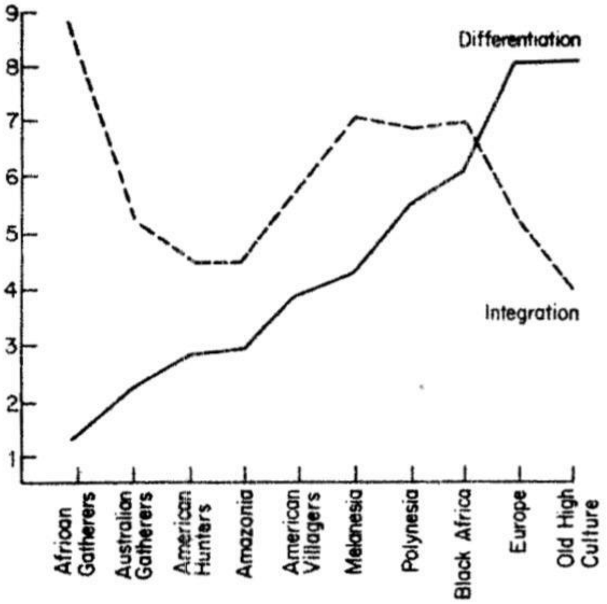

Fig S1. Covariance of song style plotted against cultural systems, from [15].

Lomax concluded that musical style is an indirect response to soecietal fundamentals, such as mode of subsistence and productivity, social organization including stratification, the organization and gendered division of labor, and climate. The covariance of the two main models driving musical style portrayed the elaboration of performance style as generally following socio-economic complexity.

S1.2.5. Independent outcomes.

Lomax et al. found that polyphonic vocal organization is most frequent in complementary socio-economies such as those of warm latitude gatherers, or gardeners with a few domestic animals, where women make an equal contribution to subsistence [17]. The

negative association between polyphony and plow agriculture has been retested [29], with higher coefficient values than those originally obtained. These findings are supported in an exhaustive study by Alesina et al [30] on the relationship between plow agriculture and women's loss of status. Consonant-Vowel syllables, which reflect a regular rhythm in language, are related to the degree of baby-holding, a theory derived from Barbara Ayres' work with Cantometrics that found regular rhythm in music significantly associated with baby-holding [28, 31]; other independent analyses have confirmed some findings. Regardless, both correlations and cluster analyses will be reanalyzed with the far more sensitive techniques now available.

S1.3. Details of the Global Jukebox datasets

The performance style project encompasses eleven primary studies of expressive behavior, two supplementary studies of social structure and subsistence strategy, and four substudies on specific aspects of social structure as they relate to song, based on hypotheses developed mainly from Cantometrics and Choreometrics data analysis. Following are brief summaries of the studies and their current status, as well as the geographic distributions of their data, and descriptions of their variables. Prior to this release, Cantometrics, Phonotactics, Choreometrics, and Parlametrics were partially published in books, films, and articles, but their coded data was not published. The other studies were never published in any form.

S1.3.1. Global Jukebox datasets included in current release

1. *Singing (Cantometrics).* Conceived and piloted by Lomax in the 1950s and completed and formalized with Victor Grauer in 1960-1962, this study considered the social organizational, integrative, and differentiating aspects of performance; vocal qualities, ornament, articulation; melodic and rhythmic characteristics and structure; and relation between vocal and orchestral parts. (Data, metadata and coding guide available for download at https://zenodo.org/record/4898406) A full training course including example and test recordings is available at http://theglobaljukebox.org after clicking on "Songs of Earth". (See Table 2 for a complete list and description of the Cantometrics variables).

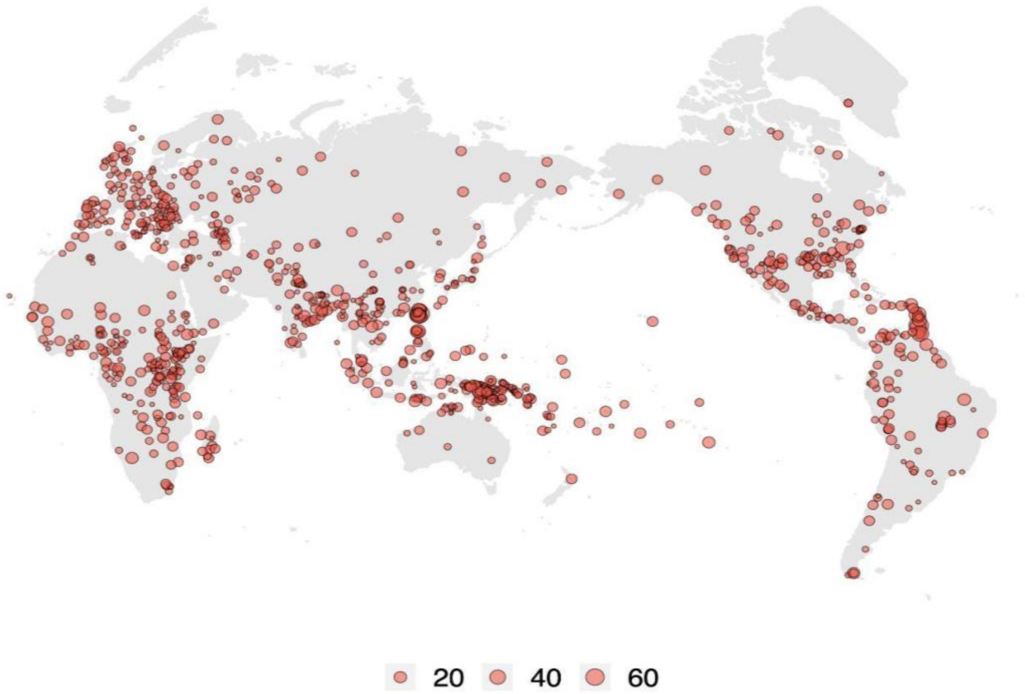

Fig S2. Location of the 1,026 societies within the Cantometrics dataset. Points are sized relative to the number of songs for each society, totalling 5,776 songs.

2. *Minutage*. This study of breath management and phrasing in indigenous and folk song investigated the ways that melody and song structure are articulated through the breath. Minutage is a way of understanding song structure as it is performed. It considers the framework of a style in terms of the habits and small decisions concerning when to end an expulsion of sound, for example, when and how long to pause, who pauses, when to let a note ring out, whether to emphasize inhalations or to cover them up. *Alan Lomax, Kathleen Mullin, Ethel Raim, Roswell Rudd, in consultation with Dr. Godfrey Arnold, Dr. M. I. Cohen, and Theodore D. Hanley.* (Data, metadata and coding guide available for download at https://zenodo.org/badge/latestdoi/343250326)

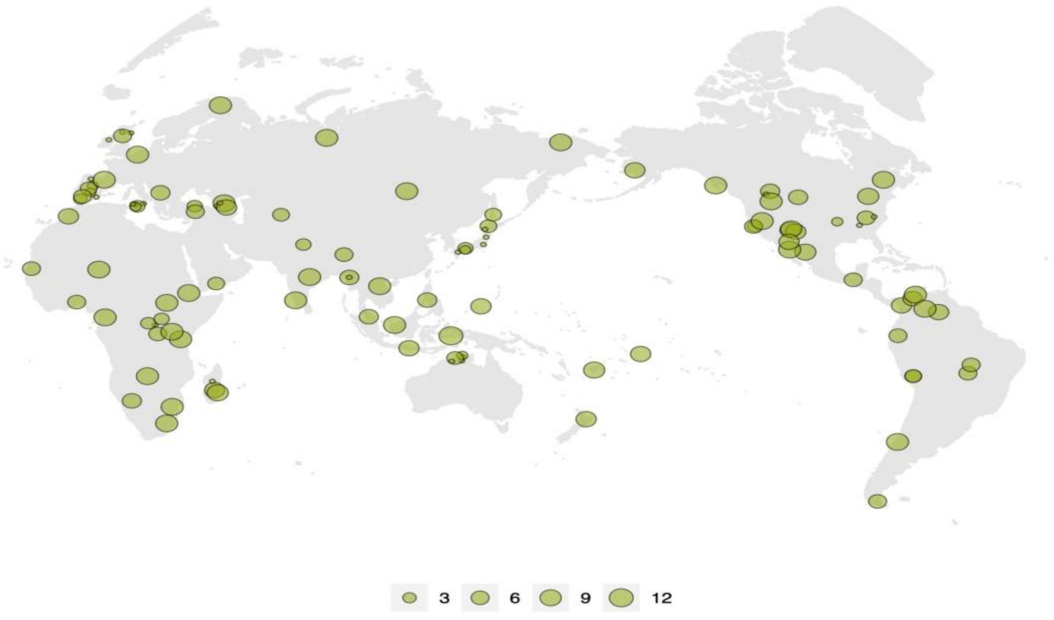

Fig S3. Location of 118 societies within the Minutage dataset who have had songs coded for breathing and phrasing in musical performance, totalling 687 recordings.

Table S3. Minutage Variables

**Line**	**Variable**	**Description**
Line 1	Gender	Gender makeup of the singing group.
Line 2A	Function 1	Primary function of the song in the context of its performance.
Line 2B	Function 2	Secondary function of the song in the context of its performance.
Line 2C	Function 3	Tertiary function of the song in the context of its performance.
Line 3	Organization of vocal group - A	Social organization of the vocal group. May be double or triple coded with Lines 4 and 5.
Line 4	Organization of vocal group - B	Social organization of the vocal group. May be double or triple coded with Lines 3 and 5.
Line 5	Organization of vocal group - C	Social organization of the vocal group. May be double or triple coded with Lines 3 and 4.
Line 6	Modal length - Leader's units	Modal length of the leader's units of phonation.
Line 7	Modal length - Chorus' units	Modal length of the chorus' units of phonation.
Line 8	Leader's % of total singing time	Percentage of total singing time allocated to the leader versus the chorus.
Line 9	Unit typology	Type of song structure based on the organization of units of phonation and pauses.

Line 10	Modal length	Modal length of the basic units of phonation, considering all components (individual singers or groups of singers).
Line 11	Range of unit lengths	Range of lengths of the basic units of phonation, considering all components (individual singers or groups of singers).
Line 12	Unit pattern	Degree to which lengths of units of phonation are organized in a pattern.
Line 13	Other patterning	Variation in length of units of phonation that might obscure patterning.
Line 14	Irregular beginning, patterned units	Situations of initial irregularity in the unit lengths of a song with patterned or regular units of phonation.
Line 15	Separate Introductions	Situations of separate introductions with contrastive melodic material.
Line 16	Terminal prolongation	Amount of prolongation of the final tone of the song.
Line 17	Sectional prolongation	Amount of prolongation of the final tone of large repeated sections.
Line 18	Coda	Situations of separate codas with contrastive melodic material.
Line 19	Orchestral Accompaniment	Presence of orchestral accompaniment.
Line 20	Presence of featured instrument	Presence or absence of a featured instrument.
Line 21	Alternate lead	Presence or absence of a second featured instrument.
Line 22	Does this instrument play the melody?	Whether the featured instrument plays the sung melody.
Line 23	Modal pauses	Modal length of pauses in the song.
Line 24	Pause structure	Compares the length of pauses between the largest repeated sections of the song with the length of large melodic units.
Line 25	Modal pause length between larger sections	Modal length of pauses between the largest repeated sections in the song.
Line 26	Number of levels	Number of melodic levels based on grouping repeated sequences of units into larger sections.
Line 27	Number of levels regular	Number of levels in which all the units are the same length.
Line 28	% Melody repeated	Proportion of the song's melody that is repeated.
Line 29	Number of units in largest repeated section	Number of basic units of phonation in the largest repeated section.
Line 30	Length of repeated section	Most frequently occuring length of the repeated sections of the song.
Line 31	Range of repeated section	Range of lengths of the repeated sections of the song.
Line 32	Amount of variation	Amount and type of variation that occurs in the repeated sections.
Line 33	Important melodic unit length	Modal length of the important melodic units of the song (defined by melodic criteria rather than pause markers).
Line 34	Range of melodic units	Range of lengths of the important melodic units of the song.

3. *Phonotactics*. A cross-cultural study of vowel and consonant placement and frequency patterns in singing, based on Lomax's observation that emotion in singing manifested in culturally specific ranges of phonological articulation by *Edith Trager Johnson, Alan Lomax, Fred C. Peng, Henry Lee Smith, and George Trage*r. (Data, metadata and coding guide available for download T https://zenodo.org/record/4898383)

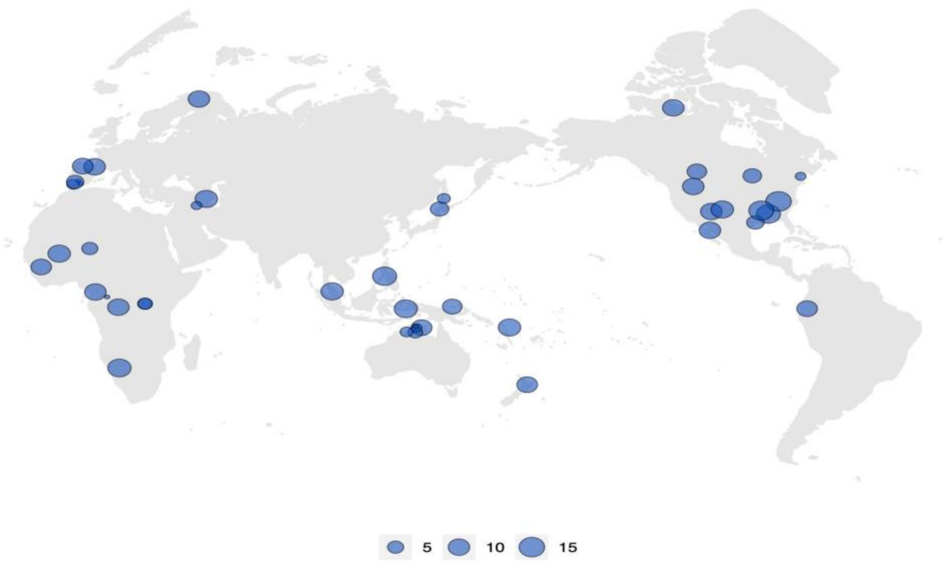

Fig S4. Location of 47 societies in the Phonotactics dataset whose songs are coded for consonant and vowel use, totalling 338 songs.

Table S4. Phonotactics Variables

**Line**	**Variable**	**Description**
Line 1	Ratio - High Front	Ratio of high front vowels to total number of vowels.
Line 2	Ratio - High Central	Ratio of high central vowels to total number of vowels.
Line 3	Ratio - High Back	Ratio of high back vowels to total number of vowels.
Line 4	Ratio - Mid Front	Ratio of mid front vowels to total number of vowels.
Line 5	Ratio - Mid Central	Ratio of mid central vowels to total number of vowels.
Line 6	Ratio - Mid Back	Ratio of mid back vowels to total number of vowels.
Line 7	Ratio - Low Front	Ratio of low front vowels to total number of vowels.
Line 8	Ratio - Low Central	Ratio of low central vowels to total number of vowels.
Line 9	Ratio - Low Back	Ratio of low back vowels to total number of vowels.
Line 10	Axis - High Front - Mid Front	Prevalence of oscillations between high front and mid front vowels.
Line 11	Axis - Mid Front - Low Front	Prevalence of oscillations between mid front and low front vowels.
Line 12	Axis - High Front - Low Front	Prevalence of oscillations between high front and low front vowels.
Line 13	Axis - High Front - High Back	Prevalence of oscillations between high front and high back vowels.
Line 14	Axis - High Back - Mid Front	Prevalence of oscillations between high back and mid front vowels.
Line 15	Axis - High Back - Low Front	Prevalence of oscillations between high back and low front vowels.
Line 16	Axis - High Back - Low Central	Prevalence of oscillations between high back and low central vowels.

Line 17	Axis - High Back - Mid Back	Prevalence of oscillations between high back and mid back vowels.
Line 18	Axis - High Front - Mid Back	Prevalence of oscillations between high front and mid back vowels.
Line 19	Axis - Mid Front - Mid Back	Prevalence of oscillations between mid front and mid back vowels.
Line 20	Axis - Mid Back - Low Front	Prevalence of oscillations between mid back and low front vowels.
Line 21	Axis - Mid Back - Low Central	Prevalence of oscillations between mid back and low central vowels.
Line 22	Axis - High Front - Low Back	Prevalence of oscillations between high front and low back vowels.
Line 23	Axis - Mid Central - Low Central	Prevalence of oscillations between mid central and low central vowels.
Line 24	Axis - High Front - Low Central	Prevalence of oscillations between high front and low central vowels.
Line 25	Axis - Mid Front - Low Central	Prevalence of oscillations between mid front and low central vowels.
Line 26	Axis - Mid Central - Low Front	Prevalence of oscillations between mid central and low front vowels.
Line 27	Axis - Mid Front - Mid Central	Prevalence of oscillations between mid front and mid central vowels.
Line 28	Axis - High Front - Mid Central	Prevalence of oscillations between high front and mid central vowels.
Line 29	Prolongation - High Front	Prominence of prolonged high front vowels.
Line 30	Prolongation - High Central	Prominence of prolonged high central vowels.
Line 31	Prolongation - High Back	Prominence of prolonged high back vowels.
Line 32	Prolongation - Mid Front	Prominence of prolonged mid front vowels.
Line 33	Prolongation - Mid Central	Prominence of prolonged mid central vowels.
Line 34	Prolongation - Mid Back	Prominence of prolonged mid back vowels.
Line 35	Prolongation - Low Front	Prominence of prolonged low front vowels.
Line 36	Prolongation - Low Central	Prominence of prolonged low central vowels.
Line 37	Prolongation - Low Back	Prominence of prolonged low back vowels.
Line 38	Ratio - Front Front Stop	Ratio of front front stop consonants to total number of consonants.
Line 39	Ratio - Front Front Fricative	Ratio of front front fricative consonants to total number of consonants.
Line 40	Ratio - Front Front Nasal	Ratio of front front nasal consonants to total number of consonants.
Line 41	Ratio - Mid Front Fricative	Ratio of mid front fricative consonants to total number of consonants.
Line 42	Ratio - Mid Mid Stop	Ratio of mid mid stop consonants to total number of consonants.
Line 43	Ratio - Mid Mid Fricative	Ratio of mid mid fricative consonants to total number of consonants.
Line 44	Ratio - Mid Mid Nasal	Ratio of mid mid nasal consonants to total number of consonants.
Line 45	Ratio - Mid Mid Lateral	Ratio of mid mid lateral consonants to total number of consonants.
Line 46	Ratio - Back Back Stop	Ratio of back back stop consonants to total number of consonants.
Line 47	Ratio - Back Back Fricative	Ratio of back back fricative consonants to total number of consonants.
Line 48	Ratio - Back Back Nasal	Ratio of back back nasal consonants to total number of consonants.
Line 49	Total Vowels	Total number of vowel areas.
Line 50	Total Consonants	Total number of consonants.
Line 51	Vowel-Consonant Proportion	Proportion of vowels to consonants.

4. ***Ensembles*** and **5.** ***Instruments*** . Two related studies of instruments and ensembles based on bibliographic information and the Sachs-Hornbostel system of classifying instruments,

which is based on how instruments were played, rather than how they were constructed. Instruments are also classified according to their gender and other symbolic functions. *Theodore Grame, Victor Grauer, Barbara Ayres, Alan Lomax, Roswell Rudd.* (Data, metadata and coding guide available for Ensembles at https://zenodo.org/record/4898378 and for Instruments at https://zenodo.org/record/4898389#.YntIR1lS_BI)

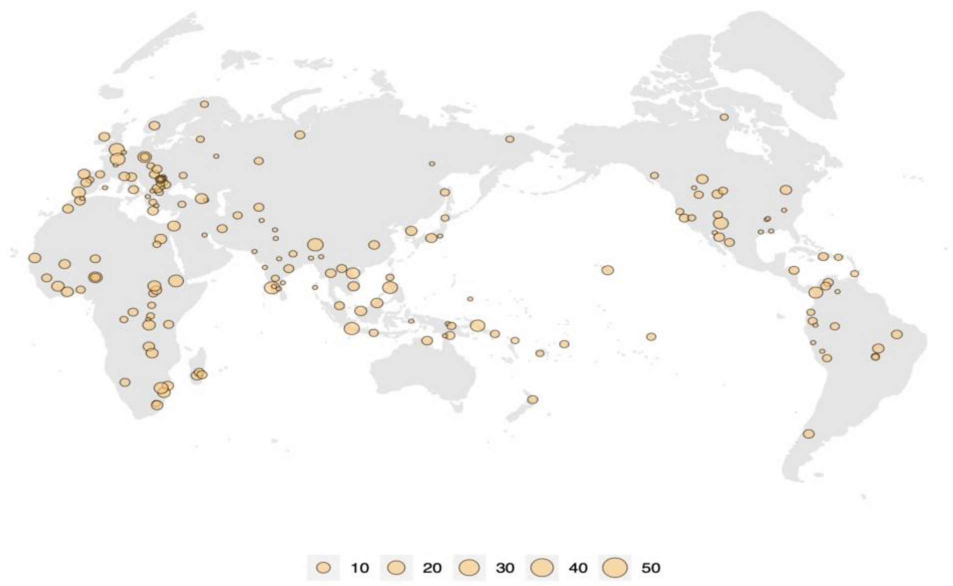

Fig S5. Location of 153 societies whose ensembles have been described, totalling 776 ensembles.

Table S5. Ensembles Variables

Line	Variable	Description
Line 1	Time Depth	Time of the ensemble's origin in the particular culture named.
Line 2	Importance of the ensemble in the culture	Prevalence and importance of the ensemble in the culture that it belongs to.
Line 3	Gender composition of the ensemble	Typical gender makeup of the ensemble.
Line 4	Female Leadership	How typical it is for the ensemble to be led by a woman.
Line 5	Number of Functions	Number of functions the ensemble has in the culture.
Line 6	Most Important Function	Most important function of the ensemble in the culture.
Line 7	Second Most Important Function	Second most important function of the ensemble in the culture.
Line 8	Third Most Important Function	Third most important function of the ensemble in the culture.
Line 9	Presence of Voices	How often the ensemble accompanies vocalists.

Line 10	Accompanying relationship of the ensemble	Relationship between the ensemble and the vocalists that it accompanies.
Line 11	Number of Instruments	Number of individual instruments in the ensemble.
Line 12	Number of Instrument Types	Number of instrument types in the ensemble.

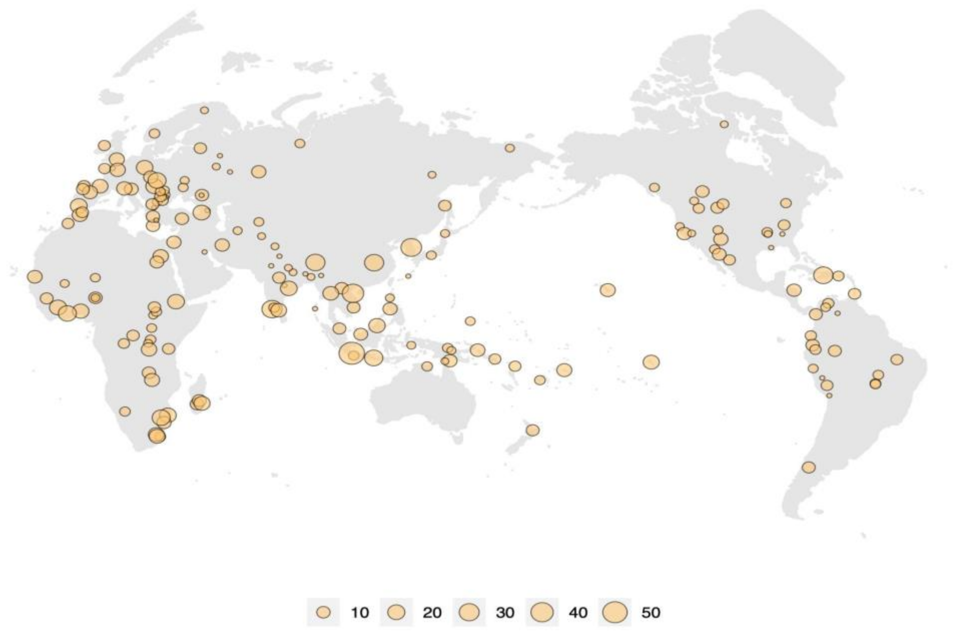

Fig S6. Location of 152 societies in the Instruments dataset whose instruments have been described, totaling 1,780 instrument descriptions.

Table S6. Instruments Variables

Line	Variable	Description
Line 1	Special info: Instrument sets; Chordo- and Aerophones	Information about instrument sets (size; tuned or untuned), chordophones (method of string activation), and aerophones (vertical or transverse).
Line 2	Special info: Membrano-, Idiophones	Method by which membranophones or idiophones are activated.
Line 3	Length	Length of the largest dimension of the instrument.
Line 4	Gender of players	Typical gender of players; instances of gendered prohibition.
Line 5	Importance of the instrument in the culture	Prevalence and importance of the instrument in the culture.
Line 6	Time Depth	Time of the instrument’s origin in the particular culture named.
Line 7	Number of Functions	Number of functions the instrument has in the culture.

Line 8	Most Important Function	Most important function of the instrument in the culture.
Line 9	Second Most Important Function	Second most important function of the instrument in the culture.
Line 10	Third Most Important Function	Third most important function of the instrument in the culture.
Line 11	Symbolism	Symbolic meaning of the instrument as understood in the culture.
Line 12	Typical Body Position	Principal body stance of the player.
Line 13	Support of the instrument	Mechanism that supports the instrument while it is being played.
Line 14	Level of resonator	Height of the instrument's resonator in relation to performer's body.

6. *Parlametrics (Conversing).* A comparative study of conversational style concerned with *how* people talk rather than *what* they say. Parlametrics regards language as living speech, a social act regulated by trans-generationally and socially transmitted conventions. By identifying such codes, tracing their variability across many societies, and observing their co-variation with key social indicators, Parlametrics seeks to uncover the metalanguage of speech, and an aesthetic framework equivalent and similar to those of movement and singing. *Norman Markel, Alan Lomax, Fred C. Peng, Norman Berkowitz, Carol Kulig and Dorothy Deng.* (Data, metadata and coding guide available for download at https://zenodo.org/record/4898385)

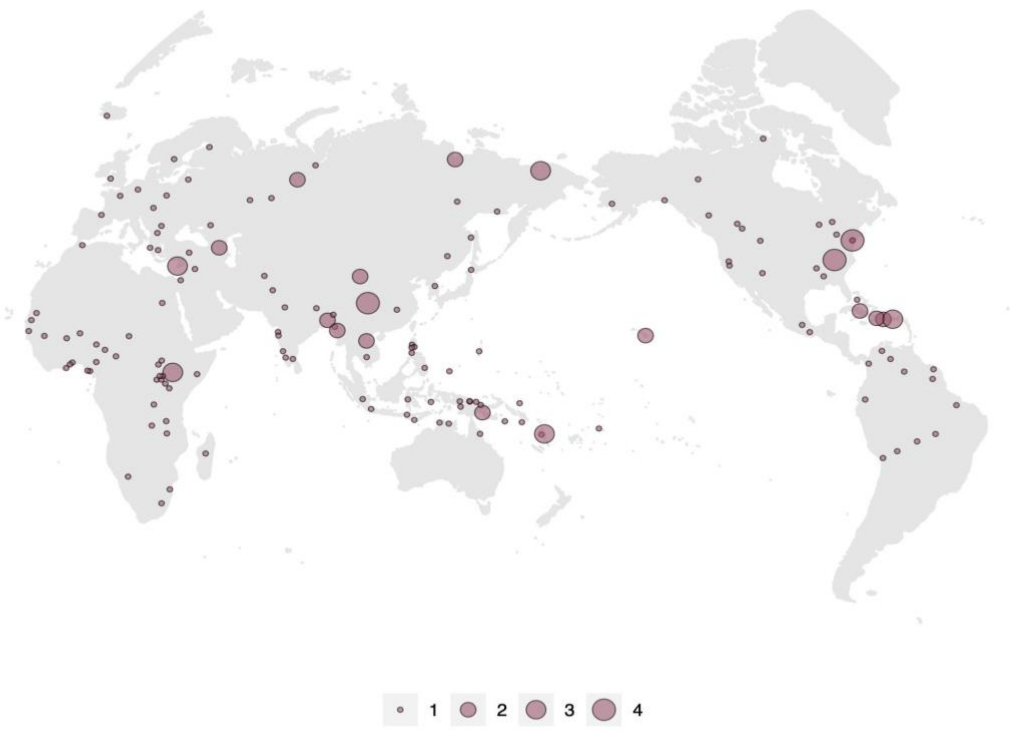

Fig S7. Location of the 158 societies in the Parlametrics dataset. Points are sized relative to the number of recordings per society, totalling 188 conversations.

Table S7. Parlametrics Variables

Line	Variable	Description
Line 1	Overall pattern of interaction	How speaking time is divided between two people in a conversation.
Line 2	Pattern of interaction in stretches	Pattern of turn-taking in stretches of conversation, regardless of which speaker assumes the leading role in each stretch.
Line 3	Speaker Similarity	Similarity of the speakers' voice qualities and dynamics.
Line 4	Principle Type of Relationship	How transitions between speakers occur.
Line 5	Stability of Role Relation	Whether speakers' relationship to each other is constant or changeful throughout the course of the conversation.
Line 6	Longest speech	Length of the longest stretch any one speaker continues, even with interruptions or interjections, before another speaker takes over.
Line 7	Number of interventions in longest speech	Number of utterances by the speaker who does not hold the floor during the longest speech measured in Line 6.
Line 8	Longest speech burst without pause/interruption	Length of the longest stretch of speech in the conversation by a single speaker, without pauses or interruptions.
Line 9A	Dominant speech burst 1	Most prominent length of speech bursts (stretches of speech by a single speaker without pause or interruption).

Line 9B	Dominant speech burst 2	Second most prominent speech burst length.
Line 9C	Dominant speech burst 3	Third most prominent speech burst length.
Line 10	Stability of Timing	Degree to which the duration of speech bursts have regular patterning.
Line 11	Attempted Interventions	Frequency of brief energetic speech bursts by one speaker during activity by another (attempts to take over the leading position).
Line 12A	Number interchanges per minute 1	Number of speaker changes during one minute of conversation.
Line 12B	Number interchanges per minute 2	Number of speaker changes during a second minute of conversation.
Line 13	Inter-speaker Transition	Degree of abruptness of the transitions between speakers.
Line 14	Gabble	Frequency of two speakers' utterances coinciding and masking each other to produce a noisy effect.
Line 15	Interjections as Responses	Frequency of interjected responses by one speaker to another.
Line 16	Murmured interjections	Frequency of quiet, brief interjections by speaker B during A's main activity that support this activity (e.g., um-hum, aha, etc.)
Line 17	Intoned Vocal Segregates	Frequency of vocal segregates with clear, musical pitch.
Line 18	Echo	Frequency with which one speaker repeats the speech of another.
Line 19	Repeats	Frequency with which a speaker repeats his or her own utterances.
Line 20	Inter-speaker Pauses	Presence and frequency of pauses between each speaker’s speech.
Line 21	Intra-speaker Pauses	Presence and frequency of pauses within the speech of one speaker.
Line 22	Vocal Stance	Degree to which each speaker's vocal stance is stable or changeful.
Line 23	Laughter	Frequency of giggling, chuckling, snickering or laughing.
Line 24	Humorous	Degree of humor and laughter-filled tone in the interaction.
Line 25	Tender	Degree of tenderness in the conversation.
Line 26	Supportive	Degree to which the relationship between the speakers is supportive.
Line 27	Excited	Level of tension and excitement in the interchange.
Line 28	Competitive	Degree to which the interchange is competitive or aggressive.
Line 29	Rich and Resonant	Presence or absence of rich resonant vocalization.
Line 30	Breathiness	Presence or absence of clear-cut breathiness.
Line 31	Harsh	Frequency of guttural, noisy and throaty sounds.
Line 32	Nasal	Frequency of a nasal tone accompanying speech.
Line 33	Hard	Absence or presence of a hard metallic quality in the voice.
Line 34	Gliding	Frequency of speakers sliding or gliding from sound to sound.
Line 35	Marked Clear Syllabification	Preponderance of short, distinct, even-spaced syllables.
Line 36	Prolongations	Frequency of drawled or prolonged syllables.
Line 37	Stress Pattern	Pattern produced by the alternation of strong and weak accents.
Line 38	Level of stress	Overall level of stress or accent in the conversation.
Line 39	Tempo	Pace or tempo of the conversation.
Line 40	Soft Volume	Frequency of markedly soft speaking volume.
Line 41	Loud Volume	Frequency of markedly loud speaking volume.
Line 42	Pitch Pattern	Degree of patterning in the pitch of the speech.

Line 43	Pitch Range	Most prominent range of pitch used throughout the speech.
Line 44	High Notes	Frequency of passages at a higher pitch than the rest of the speech.
Line 45	Descending cadence	Absence or presence of phrases in which the pitch descends.
Line 46	Upglides	Absence or presence of quick final rises in pitch at the end of phrases.
Line 47	Prolongations	Absence or presence of prolongations at the end of a phrase.
Line 48	Softening	Absence or presence of softened volume at the end of a phrase.
Line 49	Clipping	Absence or presence of phrases that end very abruptly.

7. *The Urban Strain.* A study of popular music and dance from the Tin Pan Alleys of North America. Relating a century of pop to older traditions of song and dance, the study pinpoints manifestations of a continuing interchange between African and European styles and specific innovations in music and dance defining each decade. The analysis coded each performance for Cantometrics, Personnel and Orchestra, and 18 descriptive variables designed to capture the innovative musical traits of 20th-century pop. The 400+ popular song cultures, which represent distinctive ethnic communities (sometimes multi-ethnic) that comprise regional music scenes, are reflective of the nature of pop music itself, and we have named them after their formative location, ethnicity, and decade, for example: ChicagoAA1940s (“AA” abbreviates African American). Although these cultures and subcultures had relatively short lives, their essence lingers on through their artistic works. The Urban Strain collection has been augmented by a selection of 727 popular songs spanning the last decade of the 19th century to the present, primarily representing Northern European, African/Afro American, and Eastern and Central European/Ashkenazi Jewish musical influences. *Curated by JR with ALCW and Don Fleming. Original Study: Alan Lomax, Forrestine Paulay, and Roswell Rudd.* (Metadata and audio for 727 songs available online on the Global Jukebox; data, metadata and coding guide for 378 songs available for download at https://zenodo.org/record/4898365; additional data in progress.)

Table S8. Urban Strain Variables

**Line**	**Variable**	**Description**
Line 43A	Prolongation - Vocal	Prominence of prolonged and/or very short notes in the vocal part.
Line 43B	Prolongation - Orchestra	Prominence of prolonged and/or very short notes in the instrumental part.
Line 44A	Syncopation - Vocal	Degree to which vocalists anticipate or delay the beat.
Line 44B	Syncopation - Orchestra	Degree to which instrumentalists anticipate or delay the beat.

Line 45	Vocal Tone	Special ways that singers may approach their vocal tone.
Line 46	Vocal Features	Special approaches to the musical treatment of the vocal part.
Line 47	Orchestral Tone	Special ways that instrumentalists may approach their tone.
Line 48	Orchestral Features	Special approaches to the musical treatment of the instrumental part.
Line 49	Orchestral Type	Special types of relationships within the instrumental group, and between instrumentalists and singers.
Line 50	Polyphonic Type Orch	Manner in which instrumentalists produce simultaneous intervals other than unison or the octave, if polyphony is present.
Line 51A	Harmonic Type - Vocal	Prominent type of harmonic relationship in the vocal part.
Line 51B	Harmonic Type - Orchestra	Prominent type of harmonic relationship in the instrumental part.
Line 52	Rasp - Orchestra	Degree of raspy, buzzy, scratchy, non-harmonic tone in the orchestra.
Line 53	Volume - Orchestra	Loudness of instrumental part.
Line 54	Tempo - Orchestra	Tempo of instrumental part.
Line 55	Orchestral Colors	Special approaches to timbre and production in the instrumental part.
Line 56	Collage Components	Presence of layered or collaged recordings, or musical quotes.
Line 57	Melodic Form - Orchestra	Form of the instrumental part of the song, considering the degree that melodic material is repeated (litany, strophe, or through-composed), the complexity of the form, and the degree of variation in each repeated section.

8. Social Factors **(supporting dataset)**. Cross-cultural codes on 38 socio-cultural variables adapted from the Ethnographic Atlas including geography, subsistence type, political structure, gender roles, kinship and family structures, land and property ownership, sexuality, games, and theology. *Barbara Ayres, Norman Berkowitz, Edwin Erikson, Conrad Arensberg.* (Data, metadata and coding guide available for download at https://zenodo.org/record/4898380)

Table S9. Social Factors Variables

Line	Variable	Description
Line 1	Climatic Zone	Latitude range of the society.
Line 2	Altitude	Elevation of the society.
Line 3	Size of Settlements	Average population of communities.
Line 4	Permanence of Settlement	Stability of the main type of settlement.
Line 5	Subsistence Mode	Technique used to extract food from the environment.
Line 6	Collecting	Degree of dependence on collecting as main food source.
Line 7	Hunting	Degree of dependence on hunting as main food source.
Line 8	Fishing	Degree of dependence on fishing as main food source.
Line 9	Animal Husbandry	Degree of dependence on animal husbandry.

Line 10	Intensity of Agriculture	Style of agriculture.
Line 11	Agricultural Product	Main agricultural product.
Line 12	Size of Animals	Principal form of domesticated animals.
Line 13	Milking	Presence of milking and types of animals that are milked.
Line 14	Metal Working	Presence or absence of metal working.
Line 15	Gender Complementarity: Main subsistence activity	Proportional contribution of men and women to primary food producing activity.
Line 16	Gender Complementarity: All subsistence activities	Compares proportional contribution of men and women averaged across all subsistence activities to the world average.
Line 17	Gender Complementarity: Crafts	Compares proportional contribution of men and women averaged across six craft activities to the world average
Line 18	Gender Differentiation	Compares the extent to which both craft and subsistence activities are done in gender-separate groups to the world average.
Line 19	Segregation of Adolescent Boys	Whether adolescent boys are separated from the family, perhaps as part of an initiation rite.
Line 20	Premarital Sex Norms for Females	Extent to which female premarital sexual activity is allowed or prohibited.
Line 21	Means of Marriage	Cost of marriage, and whether it falls on the groom's or the bride's family.
Line 22	Type of Polygyny	Presence or absence of polygyny; degree to which wives are separated.
Line 23	Family Size	Typical size of families that dwell together.
Line 24	Marital Residence	Which side of the family married couples reside with.
Line 25	Lineages	Mode in which descent is reckoned.
Line 26	Unilateral/Bilateral	Whether descent is calculated from one side of the family or both.
Line 27	Land Ownership	Presence or absence of land ownership.
Line 28	Inheritance of Real Property	Whether land is inherited by matrilineal heirs, patrilineal heirs, or both.
Line 29	Inheritance of Moveable Property	Whether moveable property is inherited by daughters, sons, or both.
Line 30	Kin Solidarity	Organization of marriage; presence or absence of localized kin groups.
Line 31	Kinship System	Extent to which names for cousins distinguish cousin subtypes and/or among other groups of kin.
Line 32	Community Solidarity	Distinguishes cohesive communities from more individualized ones.
Line 33	Social Layering	Additive score that considers class, caste, and slavery.
Line 34	Presence of States	Presence and size of a state system that governs the society.
Line 35	Extra-Local Jurisdictional Hierarchies	Number of non-local levels of political authority.
Line 36	Political Succession	Type of succession to the office of local headman.
Line 37	High Gods	Religious beliefs about high gods.
Line 38	Types of Games	Types and presence of games.

S1.3.2. Performance Metadata

Metadata for individual performances (recorded songs in Cantometrics, Phonotactics, and Minutage; recorded conversations in Parlametrics; bibliographic sources in Instruments and Ensembles) is summarized in Table S10.

Table S10. Performance Metadata

Dataset/Type of Data	Metadata
Cantometrics, Phonotactics, Minutage (songs)	**Internal identifiers**: Unique coding ID number; Source society ID number; Audio file ID numbers in the format used for ACE's digital collection as well as for the source tape or record in the Lomax archive; Source ID tag, which links each song to a full citation of the associated source material<br><br>**Geographic information:** Location and geographic coordinates of source society; recording location if different; location and geographic coordinates of homeland (for immigrant societies)<br><br>**Song information:** Song Name ("title"); Genre; Duration; Song Notes on content and context of the performance (summarized or quoted from field notes when available); Number, genders, and names of performers if available; Names and numbers of instruments including voice; Lyrics (in progress); Description and commentary on the performance and/or society by source society member transcribed, audio or video (in progress)<br><br>**Source Recording information:** Recordist name(s); Year recorded; Publisher; Publication or collection; Repository; Notes on the type and quality of the media file if relevant
Parlametrics (conversations)	Applicable information listed above plus:<br><br>**Language and dialect names** for both subject and researcher<br><br>**Researcher's institutional affiliations** and **details of the project** for which the speech samples were originally recorded.
Instruments and Ensembles (bibliographic data)	**Internal identifiers**: Unique coding ID number; General instrument type ID number (for instruments dataset only); Source society ID number(s) (if there is a match in our societies dataset); Source ID tag<br><br>**Instrument/Ensemble Information:** Instrument or ensemble name and alternative names; Sachs-Hornbostel instrument classification numbers (for instruments set only); List of instruments in the ensemble with corresponding instrument coding identification numbers (for

	ensembles dataset only); Specific cultural focus, if listed; Additional comments or notes from the coder<br><br>**Bibliographic Source information**: Bibliographic source citation; source tape or record number in the Lomax archive if the source is liner notes

S1.3.3. Global Jukebox Datasets In Progress

Table S11. Global Jukebox Datasets: In Progress and Derivative (In Progress: Downloadable in Future (provisional figures)

Dataset	Description	Variables	Performances / Cases	Societies	Geographic Range
**8. Choreometrics**	Movement and dance: body parts articulation, spacing form and design, body presentation, dimensionality, gender, organization	139	512 dances/ work movements	368	World
**9. Personnel & Orchestra**	Size and composition of performing groups/orchestras	15			World
**10. Song Texts**	Main themes and preoccupations appearing in song texts.				
**11. Vocal Qualities**	Indicators of the emotional content of singing				WorldF
**Popular Songs (extension of 7. Urban Strain)**	Augmentation of Urban Strain (not yet coded)	18	727	458	Regional
**Supporting Datasets**					
**Arensberg/ Lomax Historical Subsistence Taxonomy**	From a taxonomy of historical subsistence types for the cross-cultural sample (1276), of which 570 are in Societies Dataset.	17	1,276	1,276 (570*)	World
**Derivative Studies**					
**Leadership Study**	Correlations between political leadership patterns in a society and the social organization of a singing group				World
**Child Rearing Study**	Correlations between rhythmic variables and phrase length and expectations for obedience				World

**Gender Study**					World
**Work, Teams & Song Study**	Correlations between work teams and song style				World

1. ***Movement and Dance (Choreometrics)***. Developed in the mid-1960s and extending to the 1980s. Investigators coded a cross-cultural sample of human dance and work movement on film using observation-based criteria. Their analysis and choice of variables based on concepts such as shape, space, geometry, sequencing, energy and connectivity between bodies adapted in part from Rudolf Laban's analytic toolkit [32]. A few were adapted from Cantometrics, such as social organization, rhythm, articulation, differentiation, and cohesiveness, adding form and design, body presentation, gender, and organization. Choreometrics' broad level of analysis brings into relief patterns of movement strongly associated with climate zones, modes of subsistence, ancient technologies, and gender complementarity. *Irmgard Bartenieff, Forrestine Paulay and Alan Lomax, with Meriam Lobel.* (Codings are partially online on Jukebox; dance films being digitized by The Library of Congress; additional metadata and coding guide pending.)

2. ***Personnel and Orchestra***. A supplementary study to Cantometrics that deals with the size and composition of performing groups and the makeup of orchestras. An orchestra is defined as any kind of music-making by more than one individual, from two sticks tapping on a barrel to a symphony. This cross cultural survey provides for a wide range of ensembles, and supplements the coded analysis of songs done in Cantometrics. In order to round out the study of song, the Personnel and Orchestra codings must be digitized. *Alan Lomax and Roswell Rudd.* (In progress).

3. ***Song Texts***. A 1965 study investigating the repetition of words and themes in song texts, using a small sample of iconic songs from six European societies. With the addition of lyrics in the original languages and in English it will be possible to extend this study or try new approaches. *Alan Lomax and Joan Halifax, using the General Inquirer System (P.J. Stone and E.B. Hunt),* Building on research from *Benjamin Colby, Pierre Maranda, and Elli Kaija Kongas*. (In progress.)

4. *Vocal Qualities*. Cantometrics actually originated with Lomax's work on voice qualities from 1955 to 1961, which investigated the relationships between the timbre of the voice and social and psychological factors, and the crystallization of aesthetic preferences in song and speech. This early study influenced the way that vocal quality is measured in Cantometrics. *Alan Lomax, Norman Markel and Paul Moses, in consultation with Dr. Godfrey Arnold.* (In progress.)

***5. Arensberg/ Lomax Pre Industrial/Post-Colonial Subsistence Taxonomy* (supporting dataset).** Factors include levels of dependence on gathering, hunting, fishing, animal husbandry, and agriculture; predominant type of domesticated animal; predominant type of agricultural product; intensity of agriculture; presence or absence of the plow; milking; games of chance; slavery; class distinctions; wooden houses; nomadism; and kin structure [33]. A detailed classification of cultures based on specific subsistence traits and levels of those traits, this study differs from the Social Factors study described above, which includes subsistence in less detail along with other factors (political, gender, kinship, etc.) not included in the subsistence type classification. *Conrad Arensberg and Alan Lomax.* (In progress.)

6. Derivative Studies (in progress). Several studies based on results of other research are also of great interest. They include Work, Teams, & Song (Stanley Udy, Lomax, Arensberg); Leadership (Robert Textor); Child Rearing (Herbert Barry III, Irvin Child, Margaret Bacon); and Gender Complementarity. *Conrad M. Arensberg, Barbara Ayres, Alan Lomax,* drawing upon Esther Boserup's groundbreaking work on women's role in agricultural production worldwide.

S1.4. Societies

S1.4.1. Societies (supporting dataset).

The Societies dataset is a catalog of the 1,275 societies linked to the performance data in all of the studies. In the Global Jukebox, we emphasize performance as an expression of shared values and aesthetics, organizing principles, and interaction patterns within a society, or culture, as encoded in Cantometric analysis. For the convenience of researchers,

the Societies dataset is designed to link the performance data to other relevant cross-cultural datasets (see 3.2).

S1.4.2. Society Metadata

Metadata for each society in the Societies dataset include: a unique identification number; geographic coordinates based on a representative location that considers the modal location of our primary data sources for each society as well as additional research using secondary sources; focal years (date a given performance was recorded); sample size (by dataset) for each culture's representative data in our collection; Köppen climate and terrain designations and code [34]; language, language family, Glottocode [35] and language ISO code; country; Pre-industrial style cluster [17]; Pre-industrial subsistence taxon [33]; identifying information for matching societies in other D-PLACE datasets [36] (Ethnographic Atlas, Standard Cross-Cultural Sample, Binford Hunter-Gatherer, Jorgensen Western North American Indian) and eHRAF [37]; the Murdock World Sampling Province [38]; and alternative culture names. Societies will also be notated by rainfall (annual and mean distribution) and gender complementarity [19].

In our society metadata we include Murdock's "World Sampling Provinces" [38] as a carefully thought out design for grouping societies, especially for purposes of statistical analysis, which many researchers still use. In the original project, modal profiles of coded singing, dancing, etc. were created and factor-analyzed under this rubric as well as under society. We have retained provinces (encompassing societies, which in turn encompass performances) (a) for the convenience of other researchers working with provinces, and (b) because these groupings have been productive for analyzing data in the past. Although nationality became broadly consonant with culture only rather recently, we added "country" as a lookup field for the convenience of visitors and researchers relying on this point of reference.

Lomax's publications sometimes grouped peoples and song styles into regions such as "Old High Culture" derived from his research. For classification purposes, more specificity was needed for the sake of a wide public looking to connect with familiar names and places. We implemented a four-tiered geographic scheme for grouping societies: Region, Division,

Subregion (Area in Murdock) and Area (Province in Murdock). Outside of this geographical system, we added Peoples, larger groupings defined by acknowledged affiliation, cultural and historical affinity, and/or language subfamily. In many cases, these geographic and ethnolinguistic groupings correspond to Glottolog linguistic classifications used by D-PLACE. In such cases, researchers can choose which groupings are most appropriate for the question at hand.

S1.4.3. Classification, addition, and modification of societies

The Ethnographic Atlas and Standard Cross Cultural Sample classified societies according to a scheme of clusters designed to facilitate statistical sampling and minimize autocorrelation [39, 40]. We adopted neutral ethnonyms for societies, replacing vestigial colonial-mercantile or derogatory names. So as not to aggregate disparate traditions under one cultural rubric, we found it necessary to narrow categories and societies in a number of cases. For example, "Martinique" in the Murdock sample encompassed all of Martinique, but our Martinican sample represents two musical traditions, and two social orders, one urban and Creolized, and one rural, and West African. We felt it was important to recognize local, or at least provincial, tradition and culture in many instances, because up until very recently these could be quite radically distinctive, often signaling unique underlying histories and social structures [41].

The Global Jukebox societies were matched to societies in the Ethnographic Atlas, Standard Cross-Cultural Sample, Binford Hunter-Gatherer dataset, Jorgensen Western North American Indian dataset, and eHRAF database by evaluating the similarity of society names, languages, geographic coordinates, and other details from primary sources such as liner notes and field notes, and linking each applicable society with a matching D-PLACE cross-dataset identification number. To facilitate accurate matches, the Global Jukebox societies were ranked in order of priority for review based on the distance between geographic coordinates for each society's constituent songs, with the concern that societies with large distances between song recording locations may have been lumped into excessively broad groupings. Approximately 400 societies were extensively manually checked after an initial round of society matching, and broken up into more specific subgroups to facilitate matching with societies in other cross-cultural datasets where

necessary. Society and performance metadata was also verified and corrected during this process. In cases where the Global Jukebox split societies that were lumped together in other D-PLACE datasets, all relevant Global Jukebox societies were matched to the lumped society; if a specific focus was listed for a society in D-PLACE, only Global Jukebox societies with the same focus were matched.

S1.5. Data history

S1.5.1. Data History

The data itself was first coded on punch cards by Alan Lomax and his colleagues at Columbia University over 60 years ago. It has undergone numerous updates onto more modern platforms over the years but the coding format remains unchanged. From 1989 to 2006 the data lived on a relational database designed by Michael Del Rio [42] who developed the Global Jukebox prototype in the 1980s. Del Rio reprogrammed the data on the mainframe computer at Columbia University, where it had lived and grown for two decades, so that it could be housed in Apple computers and developed an interactive audio visual interface.

For the first time it was possible for a user, without the mediation of a programmer, to view and cross-reference performance datasets through the society classification hierarchy that the project had adapted from the Ethnographic Atlas. Conceptually, it was an easy jump from this to the Global Jukebox concept. One or multiple programmers could now organize and arrange data to the linking of the data controller. Code for the Jukebox was then written in refined C++. Special "tours" were created on Hypercard illustrating some of the main findings of Cantometrics, for example the "Old High Culture" style region (now called Old Eurasian), and training programs for Choreometrics, Cantometrics, and Parlametrics.

In 2006, software developer and designer Richard Smith joined the Association for Cultural Equity to transfer the Global Jukebox software and its data from the now obsolete Apple computers. With funding from the National Endowment for the Humanities, and with considerable detective work to unpack the coding format, Smith decoupled the data from the hardware and converted the 1980s Atlas to a tree structure in JSON. As the oldest

legacy code Smith and developer John Szinger had ever worked on, it presented an interesting challenge. Once the system was understood it was exciting to see the coding display come to life.

S1.5.2. version history of the cantometrics dataset

The 5,776-song Cantometrics dataset we are publishing represents a substantial expansion from the 2,527-song set that was originally analyzed, but not released, in Lomax 1968 [20], a subset of ~1,800 songs from 148 societies used in previous Cantometric analyses [4, 15, 17], and the sample of approximately 4,000 songs from 400 societies mentioned in some of Lomax's writing [43]. These data have not been released to the public until now.

The first 4,062 songs in the sample were all recorded before 1973, while the remaining ~2,000 songs were added after 1991. In 2007 Victor Grauer added 800 coded songs from hunter gatherer societies and Taiwanese indigenous peoples that had been included in a project investigating the prehistoric migration of song styles and genes from Taiwan into Oceania using Cantometrics [44-46]. Miriam El Hajli added 50 Latin American folk songs which are now being coded. Many of the newly added songs are from societies represented by one or only a handful of songs. We are including all available data to allow researchers to access and analyze the sample in ways appropriate for their purposes--focusing on sub-samples with larger numbers of performances per society, or using higher-level groupings for analysis and including songs from societies with small samples.

S1.6. Data normalization

S1.6.1. Scaling and multiple coding

Each song in Cantometrics is coded for each of the 37 variables using a single number. Scales for each variable range from 1 to 13, although in most cases do not contain all values in between. It is also possible for a song to have more than one code for a particular feature. For example, if two different extremes of volume were present in the same song (i.e. very loud and very quiet singing) both traits would be coded for the volume variable. At the time data was collected, computational constraints and the database design meant data was restricted to holding one value per feature per song. For this reason, all values were

originally coded in an exponent-based system, creating a single unique value for any combination of values within the 1 – 13 scale. The exponent system means that any single value is stored as 2 to the power of the value (2^{value}), if multiple codes exist for a feature then it is the sum of each exponentiated value. For example: a value of 3 is stored as 2^3 = 8, if the feature contains an additional value of 6 the stored value is $2^3 + 2^6 = 72$. This system of storing data is the raw format for Cantometrics, Parlametrics, and Urban Strain, and is presented for posterity, but not recommended for modern analysis. For the analysis of individual variables, we recommend converting the data back to the original 1-13 scale, using the process described below. For analysis comparing variables, we recommended converting the 1-13 scales to a 0-1 using the process described below.

Since each stored value has a unique solution which can be determined by values between 1 & 13, we can obtain the values on the original scale through an iterative process. We give some pseudo-code below to show the process using the stored value, and the set of possible values between 1-13 for the features in question:

```
-- input: Cantometrics stored codes as a sum of powers of two
-- output: Cantometrics coding values as an array of discrete
integers

-- Algorithm
S: array of integers
remainder <- input
while remainder != 0 do
      code <- floor(log2(remainder))
      remainder <- remainder - pow(2, code)
      S.insert(code)
end while

if S.length >4 do
      print("Invalid code, there can be only three codings")
      break
end if
return S
```

Data stored in the exponent system are difficult to analyze and understand, so for Cantometrics, we have additionally stored the data in the Cross-linguistic Data Format [47]. This format contains the data in the original 1 – 13 values, and in long format, which allows for multiple codes per feature per song. This can be found within the cldf/ folder of the Cantometrics repository and is the recommended dataset for future Cantometrics analyses. From the 1 - 13 values, data can then be converted using conventional standardization techniques.

Since each variable exists on a 1 – 13 scale, but the intervals of each scale vary, each variable needs to be re-ranked to a standardized scale to be comparable. To convert variables to a standardized scale, convert the 1 – 13 codes within each scale (recorded within etc/codes.csv of the Cantometrics data repository) to a linearly increasing scale (e.g. convert a scale of 1, 4, 6 etc. to 1, 2, 3 etc.). Once data is on a linear scale, divide the scale by the maximum value, to create a 0-1 scale that is equivalent across variables.

S1.6.2. Correcting coding criteria for CV-30 and 1

While the original Cantometrics coding instructions for CV-30 (Tremolo) specified a 3-point scale (Much tremolo, Some tremolo, or Little/no tremolo), and the hand-written coding sheets were coded according to this scale, the digital codings clearly showed the use of a 5-point scale (Much tremolo, Moderate tremolo, Some tremolo, Slight tremolo, Little/no tremolo). Documentation of this change, which may have occurred during the digitization process, was not found. The coding instructions and other materials referencing CV#30 on the Global Jukebox were amended to reflect the 5-point scale used in the current data.

The original version of Cantometrics included two different codings of "solo singer" for CV-1:

2 ("One singer, whether accompanied by instruments or not"), and 3 ("One singer with an audience whose dancing, shouting, etc. can be heard. In practice we omitted this point and coded all solos '2'"). To make the codings consistent with this description, we recoded 2 songs coded as "3" for CV-1 as "2".

S1.6.3. Data exclusion

Prior to publication, we removed audio recordings containing only instrumental music without vocal song, since Cantometrics was intended to measure and compare songs, not instrumental music. These codings had been originally coded with the idea of potentially expanding the method/sample to include instrumental music, but this was never followed through systematically. To do this, ~100 samples coded as "missing data" or "no singers" for CV-1 (Cantometrics Variable number 1) were excluded. We also excluded any popular songs added to the database, in order to ensure consistency with the original sampling criteria of restricting samples to traditional songs. Finally, we excluded 107 songs that were only partially coded (missing codings for 7 or more variables).

S1.6.4. Correcting "impossible codings"

Around 30 impossible coding values--codes that did not correspond with a defined scale point--were identified and corrected. Digitization errors accounted for all of the impossible coding values that were found amongst songs coded in the original sample, and these were corrected by referring to the original hand-written Cantometrics coding sheets. Nineteen out of the 30 impossible codes were associated with songs that had been digitally coded in a later addition to the Cantometrics sample, with no hard copies to reference in order to identify the source of the errors. In these cases, ACE staff listened to the recordings and re-coded the appropriate variables.

S1.6.5. Missing, incomplete or poorly recorded audio files

Over 1,200 audio files were missing and many others were curtailed or of poor quality. In collaboration with the Curator of the Alan Lomax Collection at The Library of Congress ACE researchers found nearly a thousand of these missing audio files on LPs and 7" tape reels at the Library of Congress, at Indiana University, and through Discogs. We also identified tracks with the worst audio problems and those with better versions, and had the worst audio tracks restored. Efforts to recover missing audio have recently resumed. Sound restoration will be resumed when possible.

S1.7. Coding reliability

S1.7.1. Inter-rater reliability

The reliability of Cantometric ratings (and of Ethnographic Atlas ratings [27]) have been a focus of previous criticism [9, 48-50; cf. [18] for discussion]. Several studies have explored inter-rater reliability of Cantometrics and related schemes for other datasets [9, 17, 45, 50-52], but none have validated the reliability of the actual dataset of codings in the Global Jukebox.

To directly examine reliability of the Cantometric dataset, PES and ALCW recoded 30 songs randomly selected from the full Cantometric dataset using the 37 Cantometric features blind to any information about the songs' metadata or their codings in the database (see https://osf.io/f57a8 for analysis preregistration and Inter-rater Reliability/GJBIRRPreReg.R in https://zenodo.org/record/6537663#.YnszmllS_BK for pre-registered analysis code). We chose to have PES and ALCW code the songs rather than someone else blind to our hypotheses because they have spent over a decade using Cantometrics and are probably the most experienced living coders other than Victor Grauer (Grauer originally co-created Cantometrics and coded many of the songs in the Cantometric database with Lomax, and thus would not be an appropriate choice to test inter-rater reliability against what may be his own codings). They coded the songs independently once, then compared their codings with one another and revised them appropriately over the course of several iterations into a combined "consensus" set of codings agreed on by both (this consensus was agreed on prior to unblinding and analyzing the data). After running the analyses, it was discovered that one of the 30 randomly selected songs was one of the uncoded songs with missing data that was eventually excluded from the dataset. This song was excluded from the inter-rater reliability analyses (note that this exclusion was not included in the pre-registration because we had not anticipated this possibility).

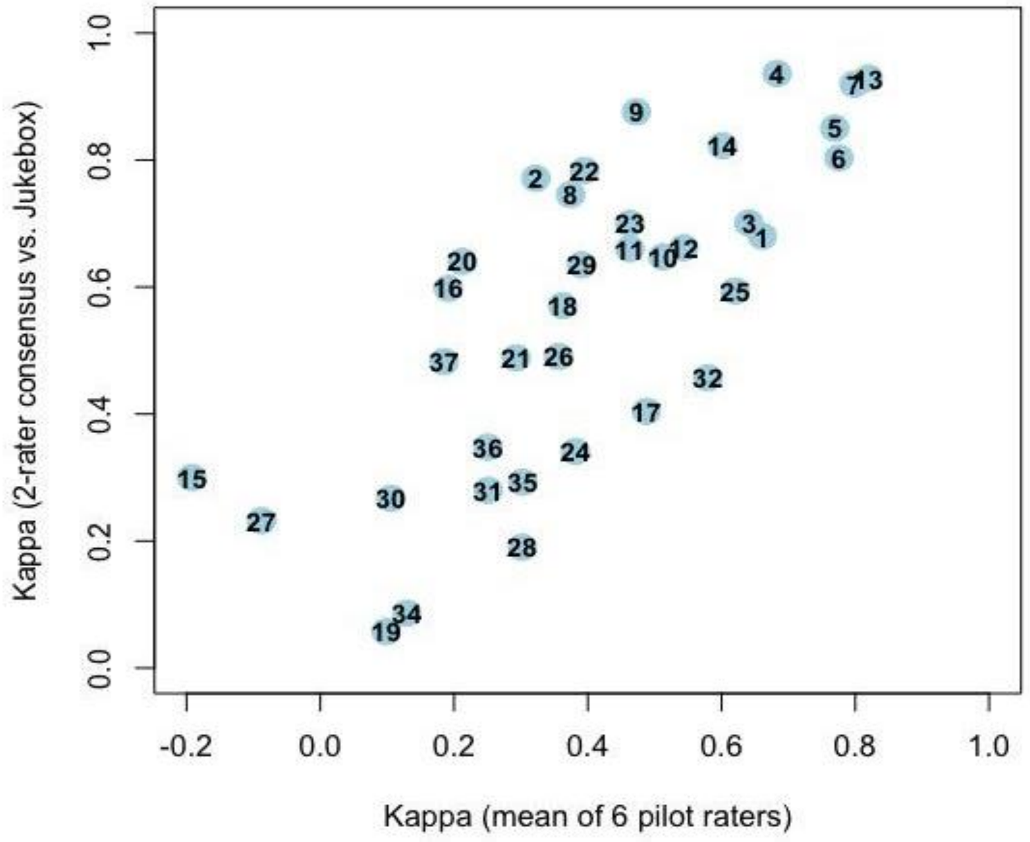

Fig S8. Correlation between inter-rater reliability values for the 37 Cantometric variables obtained comparing ALCW and PES's consensus codings with the Global Jukebox Cantometrics codings (y-axis) with pilot analyses based on training data from 6 members of the CompMusic Lab (x-axis). The values were strongly correlated (r = .72), but with ALCW and PES's values consistently higher than the training codings (mean K = .54 vs. 0.40, respectively).

Table S12 shows the results of the inter-rater analysis. As predicted in our pre-registration, mean reliability for all 37 Cantometric variables was significantly greater than chance (mean K = 0.54, t = 12.5, df= 36, p = 6.3×10^{-15}) and our results with the Cantometric dataset were significantly correlated with our pilot analyses based on a dataset of training codings by 6 members of the CompMusic Lab (r = .72, df = 35, p=6.5×10^{-7}; Fig S8). While there is no general agreement about interpreting K values, our observed mean value of .54 has been described as "moderate" [53] and is higher than the threshold of .4 recommended as a minimum for clinical applications [54, 55]. It is also higher than the mean level of 0.40 found in our pilot analyses using a different set of 30 songs coded independently by 6

members of the Keio University SFC CompMusic Lab, and higher than mean levels ranging from 0.24-0.47 found in similar analyses using Cantometrics or related schemes [9, 45, 51, 52]. (NB: Mehr et al. [9] did not report Kappa statistics, but when their publicly available analyses are reproduced using Light's Kappa [i.e., the average of all pairwise Cohen's Kappas, implemented by replacing "psych::alpha" with "psych::cohen.kappa" at https://github.com/themusiclab/nhs/blob/master/analysis/script4_analyze_disco_final.R CV181] instead of Crombach's Alpha, their observed mean Kappa value is 0.24).

The mean when comparing ALCW and PES's combined consensus coding with the Cantometric dataset was substantially higher than the mean when comparing ALCW and PES's individual codings prior to the consensus agreement with one another and the dataset (K=0.54 vs. 0.46, respectively). The higher reliability observed here thus appears to reflect both the benefit of ALCW and PES's depth of experience coding songs around the world using Cantometrics and the benefit of combining two independent codings (of PES and ALCW) into a single consensus coding, which previous studies did not do, but which follows the practice of collaborative coding originally employed by Lomax and Grauer. Overall, these analyses allow the reader to assess the level of confidence they should have in individual codings/variables. Of course, combined consensus codings still reflect a certain degree of subjectivity, and researchers are advised to familiarize themselves deeply with the online coding manual and example recordings ("Songs of Earth" at https://theglobaljukebox.org/?songsofearth) and take into account the complexities, subjectivities, and nuances of this manual coding scheme when analyzing and interpreting Cantometric data.

While all Cantometrics variables were intended to represent an ordinal continuum, some are more ordered than others. For example, Lines like 4/7 (Musical organization of the voice/orchestra) could arguably be more appropriately treated as categorical (unordered). This treatment does not have major effects for the current reliability analyses - while weighted Kappa was used to treat all variables as if they were ordinal, the effect of variables not being truly ordinal will simply be for the resulting Kappa values to decrease to what they would have been if treated as unweighted (categorical). However, this issue should be

kept in mind for future analyses when such treatment might conceivably impact the analyses.

Table S12. Inter-rater agreement statistics for each Cantometrics variable

		Consensus (ALCW&PES) vs. database			Mean of individual ratings (ALCW, PES, and database)		
**Variable number**	**Variable name**	**κ**	**% agreement**	**r**	**κ**	**% agreement**	**r**
1	The social organization of the vocal group	**0.68**	52	0.87	0.6	49	0.91
2	Relationship of orchestra to vocal parts	**0.77**	64	0.69	0.55	64	0.29
3	Social organization of the orchestra	**0.7**	69	0.7	0.78	73	0.82
4	Musical organization of the vocal part	**0.94**	89	0.94	0.92	82	0.94
5	Tonal blend of the vocal group	**0.85**	52	0.86	0.78	51	0.79
6	Rhythmic coordination of the vocal group	**0.8**	55	0.81	0.73	47	0.79
7	Musical organization of the orchestra	**0.92**	86	0.95	0.83	76	0.82
8	Tonal blend of the orchestra	**0.75**	69	0.75	0.68	67	0.69
9	Rhythmic coordination of the orchestra	**0.88**	72	0.88	0.7	72	0.78
10	Repetition of text	**0.65**	41	0.67	0.42	26	0.53
11	Overall rhythm: vocal	**0.66**	69	0.69	0.35	50	0.24
12	Rhythmic relationship within the vocal group	**0.66**	70	0.74	0.58	57	0.6
13	Overall rhythm: orchestra	**0.93**	83	0.9	0.87	69	0.89
14	Rhythmic relationship within the orchestra	**0.82**	75	0.85	0.6	70	0.62
15	Melodic shape	**0.3**	50	0.41	0.19	42	0.35
16	Melodic form	**0.6**	32	0.6	0.61	33	0.62

17	Phrase length	**0.4**	26	0.46	0.45	38	0.52
18	Number of phrases	**0.57**	21	0.56	0.48	33	0.48
19	Position of final tone	**0.06**	34	0.09	0.06	30	0.16
20	Melodic range	**0.64**	71	0.64	0.38	46	0.37
21	Interval size	**0.49**	55	0.52	0.47	36	0.6
22	Polyphonic type	**0.78**	72	0.8	0.6	61	0.75
23	Embellishment	**0.7**	45	0.7	0.65	48	0.66
24	Tempo	**0.34**	45	0.4	0.52	52	0.47
25	Volume	**0.59**	46	0.65	0.51	44	0.56
26	Rubato: vocal	**0.49**	45	0.5	0.47	42	0.65
27	Rubato: orchestra	**0.23**	86	0.2	0.02	77	0.11
28	Glissando	**0.19**	52	0.21	0.14	47	-0.1
29	Melisma	**0.63**	69	0.67	0.45	53	0.61
30	Tremolo	**0.27**	17	0.4	0.2	30	0.35
31	Glottal	**0.28**	62	0.31	0.14	56	-0.1
32	Vocal pitch (register)	**0.46**	41	0.49	0.23	41	0.4
33	Vocal width	**-0.1**	27	-0.12	0.02	28	-0.1
34	Nasality	**0.09**	8	0.26	0.21	24	0.51
35	Rasp	**0.29**	23	0.31	0.22	26	0.33
36	Accent	**0.35**	45	0.35	0.25	38	0.49
37	Enunciation	**0.48**	46	0.49	0.26	32	0.38
**Mean**		**0.54**	53	0.57	0.46	49	0.51

We show the average values across three comparisons within the two raters [ALCW and PES] and the database (right), and the values obtained between the consensus [ALCW and PES combined] and database ratings (left). We show for each case, from left to right: Cohen's κ, % agreement, and Pearson's correlation coefficient. Cohen's Kappa is an inter-rater reliability metric, ranging from -1 to 1. 1 indicates perfect agreement, and 0 indicates matches are occurring at random. Bold Kappa values for the consensus codings indicate that these were the focus of our pre-registered predictions; other statistics are exploratory.

S1.7.2. Inter-rater accuracy

Any measurement comes with an associated error. While the Kappa statistic is useful as a measure of how well raters agree with each other, it falls short of an error measurement. For example, it is possible to have high accuracy, yet low Kappa, and vice versa. In Fig S9 we show the percentage agreement as a simple measure of accuracy, and how it compares to Kappa. For the purposes of propagating errors in downstream analyses, the standard deviation is more useful. To calculate the standard deviation of a Cantometrics variable, we first reduce each variable to an integer scale with no gaps, and then compare individual ratings for each song to the average rating for each song. In Fig S9, we can see that the % agreement and the standard deviation do not have an exact inverse relationship. This happens, in part because not all of the variables are strictly ordinal, and also because some variables have larger deviations due to having a greater range of responses (16 - Melodic Form has 13 possible responses; 30 - Tremolo has 3 possible responses); the latter case, if problematic, can be corrected for by dividing the standard deviation by the number of possible responses.

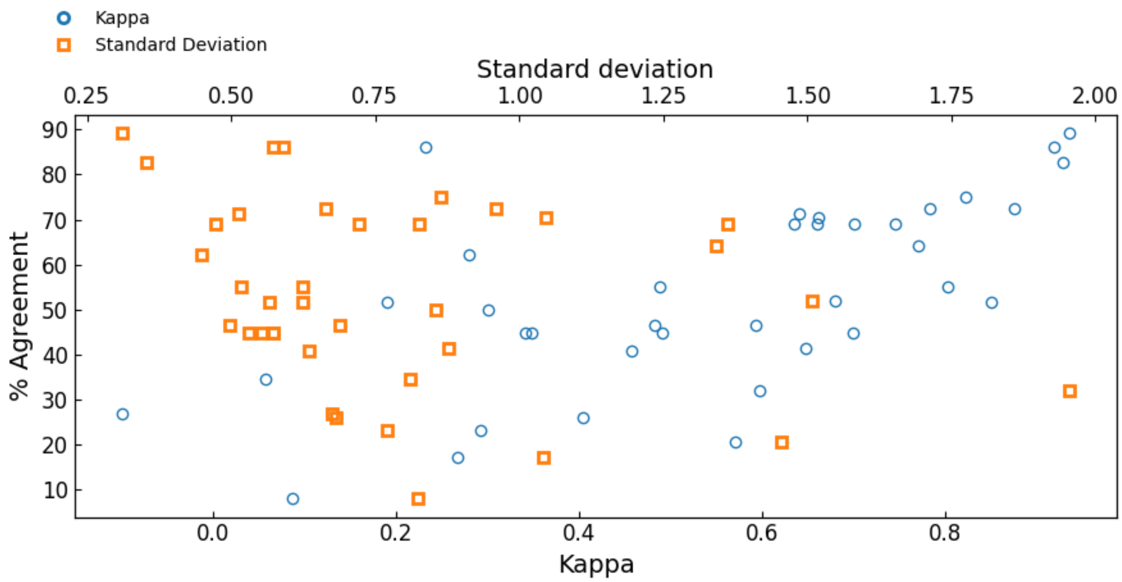

Fig S9. Inter-rater % agreement as a function of Kappa (bottom axis, blue) and the standard deviation (top axis, orange).

S1.8. Data validation

S1.8.1. Overview

We tried three separate, objective approaches to evaluate the reliability of the Cantometrics data set. We first note the proportion of recordings for which codings are absent. We then apply computational tools to assess Cantometrics criteria such as the inclusion or absence of instruments / number of singers. Finally, we exploit the few instances of redundancy in the Cantometrics system to check for consistency.

S1.8.2. Missing codings

For each recording, there should be at least one value coded for each variable. Upon checking the data, we found that 300 out of 5,776 (5%) of recordings are missing at least one variable (Table S13). In total, out of 5,776 songs and 37 variables, only 408 out of 213,712 codings (0.2%) are missing. These can be added in future releases by listening to and recoding the audio.

Table S13. Frequency of recordings that are missing # number of variables, and the corresponding totals

**# Missing variables**	**1**	**2**	**3**	**4**	**5**	**Total**
**# Recordings**	222	57	14	5	2	300
**#Total missing variables**	222	114	42	20	10	408

S1.8.3. Comparison with computational analyses

We compare Cantometrics codings with results from computational analyses in such a way that we can list the recordings in order of how likely they are to have errors. We then manually checked the items on the list, starting from the error-prone side. We calculated a moving average (window size of 20) of the fraction of recordings with errors (Fig S10). This average decreases approximately linearly with respect to the number of recordings checked. Thus, we consider that we have found the majority of each type of error when the moving average hits zero.

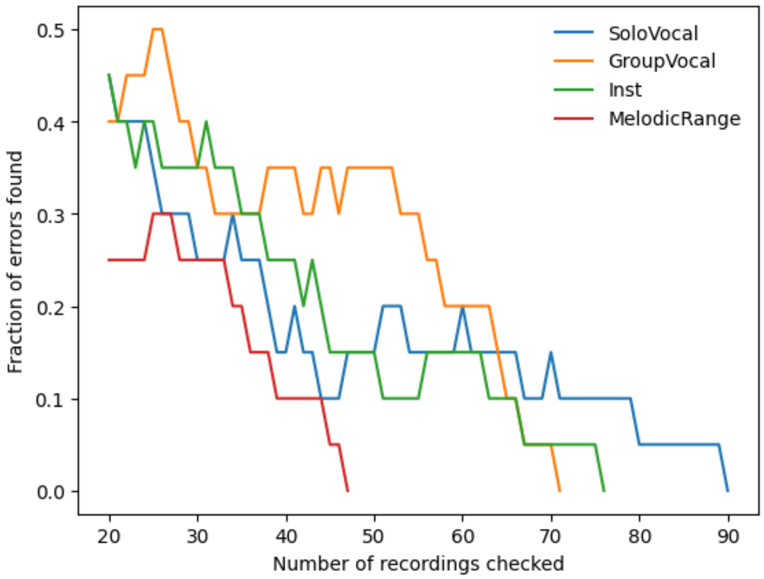

Fig S10. Moving average of the number of errors found against the number of recordings checked. A window size of 20 is used.

We use a neural network (NN) classifier that distinguishes between speech, solo singing, group singing, and instrumental [56]. Using this classifier, we estimate what fraction of each recording fits into these four categories. We then separately identify recordings as Solo Vocal (SV; no instrument), Group Vocal (GV; no instrument), and Contains Instrument (CI; can also include singing) according to the Cantometrics codings shown in Table S14. For SV recordings, we list recordings starting with those that are estimated to have the smallest fraction of solo singing. Likewise for GV / CI we start with those estimated to have the smallest fraction of group singing / instrumental music.

We also use the fact that the human vocal range is typically constrained to about two octaves. This enables us to check for potential errors in recordings that are labeled SV according to Cantometrics codings. We use the pYIN algorithm to estimate the fundamental frequency throughout the recording [57], and list those recordings that are labeled monophonic vocal in descending order of the estimated melodic range in cents.

We manually checked and corrected the errors by referring to the original hand-written coding sheets as well as to the metadata and source audio, to identify whether the error occurred during the digitization process of the data, metadata, or audio. Digitization errors in the data were corrected by changing the codes to match those on the hand-

written sheets, and mislabelled, incorrect, or incomplete audio was corrected by retrieving the correct audio from the Library of Congress and other sources, and updating the metadata where necessary to reflect this. Songs that were coded during a later addition to the Cantometrics sample and did not have hard copy coding sheets, but whose metadata and audio appeared otherwise correct, were manually re-coded by ACE staff.

The NN classification algorithm appears to have incorrectly labeled recordings largely due to bad quality recordings. However there are certain types of singing that are under-represented in the data used to train the NN algorithm [56]. As a result, the algorithm sometimes incorrectly labels old singers and chant singing as speech, female singers are sometimes labeled instrumental. Additionally, the NN algorithm cannot handle overlapping categories, so soft sounds like clapping can be ignored when heard along with singing. The pYIN algorithm flagged many recordings as having a large melodic range, but the cantometrics codings were typically correct. These erroneous flags were mostly due to background noise / poor quality recordings, or when a male speaker introduces a female singer.

Table S14. Recording categories, their definitions according to Cantometrics codings and computational measures

Recording categories	Coding logic	Computational measure	Recordings checked	Errors found
Solo Vocal (SV), no Instrument	CV1-2 & CV3-1 & CV4-4 & CV7-1	NN classifier: fraction of song classified as solo singing	90	17
Group Vocal (GV), no Instrument	(not CV2-1) & CV3-1 & CV4-4 & CV7-1	NN classifier: fraction of song classified as group singing	71	19
Contains Instrument (CI)	(not CV3-1) & (not CV4-4) & (not CV7-1)	NN classifier: fraction of song classified as instrument	76	17
SV2; Melodic Range	CV2-1 & CV3-1 & CV4-4 & CV7-1	PYIN f0 estimation: melodic range (cents)	47	7

The numbers of recordings checked and errors found are equivalent to those shown in Fig S10. Coding logic values means Cantometrics Variable (CV), the following value is the number of the variable in question (1-37), followed by the code in question. e.g. CV2-1 is Cantometrics variable 2, code 1.

S1.8.4. Consistency of codings

Cantometrics has some degree of redundancy – e.g. there are multiple codings which indicate no instrument – which allows us to check the codings for consistency. Codings CV2-1 & CV3-1 & CV7-1 & CV8-1 & CV9-1 & CV13-1 & CV14-1 must all be present if there is an instrument heard on the recording; CV2-1 & CV3-1 & CV13-1 must all be absent if there is no instrument; we found 49 instances where this was not true. Codings CV4-4 & CV5-1 & CV12-1 must all be present if singing is monophonic; CV4-4 & CV5-1 must all be absent if there is any non-monophonic singing; we found 70 instances where this was not true. Due to codings related to polyphony, if CV22-1 is present, then CV4-13 & CV16-13 must be present; if CV22-1 is absent then CV4-13 must be present; we found 179 instances where this was not true. The Cantometrics guide also specifies that if CV11-13 is present then so must CV26-1, and if CV13-13 is present then so must CV27-1; we found 181 instances where this was not true.

In total we found 349 instances of inconsistency in codings. Corrections are in progress and will be uploaded to Zenodo in new versions of the codings.

S1.8.5 Summary

Through a comprehensive screening process we identified several types of errors in the Cantometrics data set. In particular, we note that 300 out of 5,776 recordings (5%) are missing one or more codings (this amounts to a 0.2% missing variable rate). A computation screening approach identified 60 recordings (out of 284) that were incorrectly labeled with respect to instruments / number of singers; while we cannot rule out more of the same type of error in the rest of the recordings, this appears to be close to the limit of the number of errors that are identifiable using this method. Finally, we found a total of 349 instances of inconsistencies between coded variables.

It is difficult, due to the various screening methods and consistency criteria, to generalize these results to the rest of the data set. What we can say is that of the errors corrected so

far, 59% were due to coding errors and 41% were due to various errors due to the digitization process. We think that these errors cover the majority of the objective errors (absence / presence of instruments / multiple singers). More errors undoubtedly remain, but they may be more subjective and harder to find. We can estimate the amount of remaining errors by assuming the rate is constant across coding variables. We managed to check 92,304 = 16 x 5,769 (the 16 variables investigated, *i.e.*, CV2-1, CV3-1, CV4-4, CV4-13, CV5-1, CV7-1, CV8-1, CV9-1, CV12-1, CV13-1, CV13-13, CV14-1, CV16-13, CV22-1, CV26-1, CV27-1) variables in a systematic way, and out of these we found 409 errors. Since we don't know if we found all of the errors in the 16 x 5,769, the resulting extrapolation is a lower bound for these variables. On the other hand, these variables may be more likely to have errors than other variables due to explicit and somewhat complex coding instructions, so the error rate in other variables may likely be lower. We therefore treat the estimated lower bound rate of 0.4% errors per variable per song identified for these variables as a reasonable estimate of the overall error rate throughout the data set, though the actual rate may be more likely to be somewhat higher than lower (we speculate that 0.4-1% may be a reasonable likely range).

Although we may never correct all errors, we have added a "Comments and Feedback" function to the website where users may report coding/metadata errors or give other feedback. This function can be accessed by clicking the icon at the top of the Cantometric coding/metadata sheet for a given song (heart-shaped icon next to the "Song Description" text near the top of Fig 2), or the 'Comments' option in the menu at the bottom of each page.

S1.9. Current full analyses: song style correlates with social complexity (controlling for autocorrelation)

S1.9.1. The Data

Data for the musical measures are taken from Cantometrics (available at https://zenodo.org/record/4898406#.Yns0NllS_BI), and data on social complexity are taken from the Ethnographic Atlas, via D-PLACE (available at d-place.org; [36, 58]). All

data cleaning and analysis scripts for this document can be found at https://zenodo.org/record/6537663#.YnszmllS_BK.

The unit of analyses in the Cantometrics dataset is songs, held within societies. Here, we calculate what was described by Alan Lomax as a *modal profile*, offering a single musical measure for each feature (variable) for each society. Modal profiles take the modal value (i.e. most frequent) for each Cantometrics feature, across all songs within a society. The result is a single value for each feature within a society, offering an overview (or profile) of a society's musical style. Modal profiles are calculated in the script generate_modal_profiles.py.

In Section 6 we used three models to test the relationship between musical style and social complexity predicted by Lomax. Here they are explained in detail. We selected to re-test five key hypotheses proposed by Lomax.

1. *The musical texture of the orchestra becomes more elaborate as extra local jurisdictional levels increase.* (Musical organization of the orchestra (CV7) vs. Jurisdictional hierarchy beyond local community (Ethnographic Atlas: EA033).
2. *Song texts are longer and less repetitive in pastoral, plow, and irrigation based economies.* Text repetition (CV10) vs. Subsistence (an aggregate variable of Ethnographic Atlas variables detailed below).
3. *Embellishment occurs most frequently with layered social structures.* Embellishment (CV23) [rev] vs. Class (EA066), Caste (EA068), and Slavery (EA070).
4. *Melodic intervals tend to be narrower where populations are large.* Melodic interval size (CV21) vs. Community Size (EA031).
5. *Enunciation of consonants is more precise where there are extra-local Jurisdictional hierarchies.* Enunciation (CV37) [rev] vs. Jurisdictional hierarchy beyond local community (EA033).

Subsistence is an aggregate variable in the Social Factors dataset, designed by the original Cantometrics anthropologists, in consultation with Murdock, to delineate five prevalent subsistence strategies based on the Ethnographic Atlas. We have been able to reconcile

several inconsistencies in the original scale (S1.9.4). The calculation of the subsistence variable occurs in the script make_embersubsistence.R. The coding for Embellishment (CV23) is reversed so that a high score means more embellishment. The coding for Enunciation (CV37) is reverse coded so that higher values indicate more precise enunciation.

Both the musical and social data is subset and standardized prior to statistical analysis. For each hypothesis, the data was subset to a set of complete data (i.e. no missing data). The count of data for each model is shown in Table S17. All musical variables are standardized to a 0 - 1 scale according to the procedure described in S1.6.1. All social variables are divided by their maximum value, meaning they also exist on a 0 - 1 scale. Data on the linguistic and geographical categorisation of societies was determined from society metadata held within the Global Jukebox. The creation of the data for the statistical models can be found in make_modeldata.R.

Note that our primary goal was to test for a correlation between musical PC1 and social PC1, so we report 1-tailed, uncorrected p-values. We also report 1-tailed, uncorrected p-values for the bivariate correlations below for comparison with Lomax's original analyses (which also did not correct for multiple comparisons). These analyses were not formally pre-registered, but they were designed to replicate and extend Lomax's previously published findings, as described in the text.

S1.9.2. Principal component analyses

We use principal component analysis (PCA) to extract the latent variation within the musical and social variables described above. Principal components are extracted to represent the latent diversity of the musical and social variables separately. For each set of variables (musical or social), we analyze a subset of the data that includes only societies who have data for all variables (i.e. no missing data), then observe the scree plot to determine a sensible number of principal components. Finally, we extract principal components for the social and musical data.

S1.9.3. Musical PCA

We perform a PCA on the musical variables: CV7, CV10, CV21, CV23, and CV37. We use the resulting eigenvalues and scree plot (Fig S11) to determine how many dimensions should be used to represent the latent diversity. Typically, an eigenvalue greater than 1 indicates that a principal component contains more variation than any single variable, also known as the Kaiser rule. The first principal component has an eigenvalue of 2.06, and the second component has an eigenvalue of 1.003. Since the second variable effectively explains the same amount as any single variable, we opt for using a single principal component to represent musical diversity. The single musical component explains 45% of diversity. Table S15 shows the loadings of each variable onto the principal component.

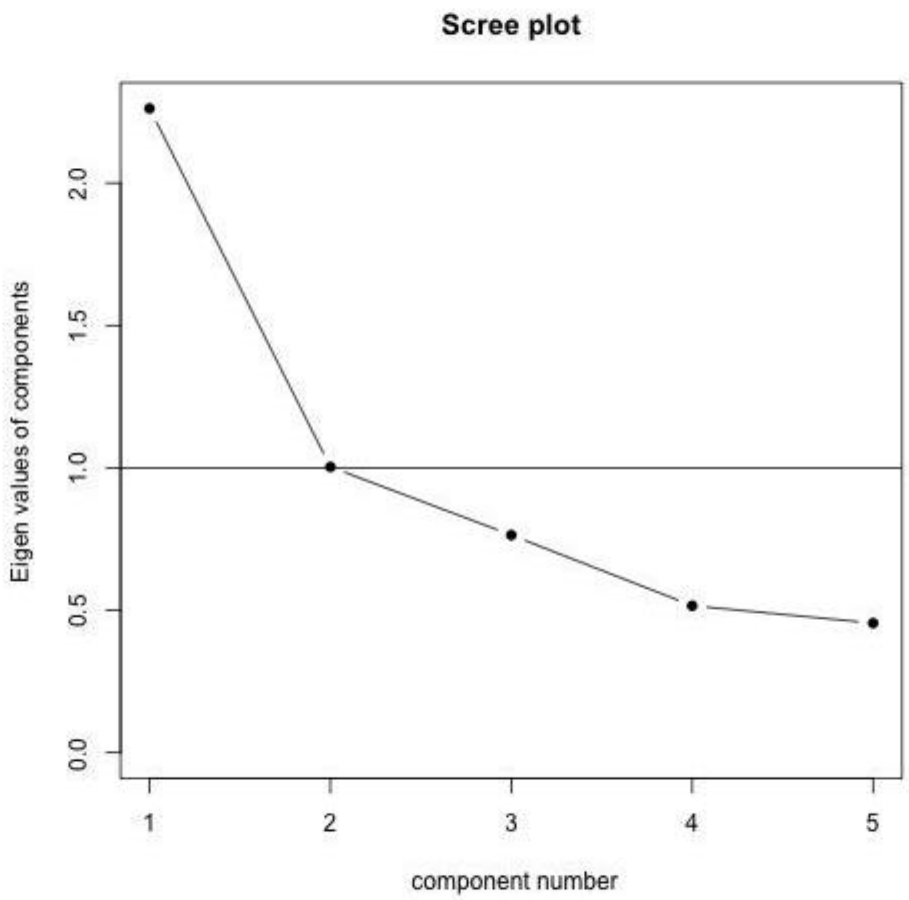

Fig S11: Scree plot of musical data, showing two components with an eigenvalue greater than 1.

Table S15: Variable loadings on the single principal component

Variable	Loading

Musical organization of the orchestra (CV7)	0.319
Text repetition (CV10)	-0.730
Melodic interval size (CV21)	-0.781
Embellishment (CV23)	0.652
Enunciation (CV37)	0.738

S1.9.4. Social PCA

We perform a PCA on the societal variables from the Ethnographic Atlas (retrieved from D-PLACE; originally from Murdock 1967): EA031 (mean community size), EA033 (jurisdictional hierarchy beyond the community), EA066 (class), EA068 (caste), EA070 (slavery), and an aggregate Subsistence variable described in Table S25. The principal component analysis estimates the eigenvalue for the first component to be 3.46, and the second eigenvalue to be 1.001. Since this variable contains only slightly more information than any single variable, we again opt to represent social diversity with a single principal component. A single principal component explains around 58% of variation. We label this principal component "societal complexity,"

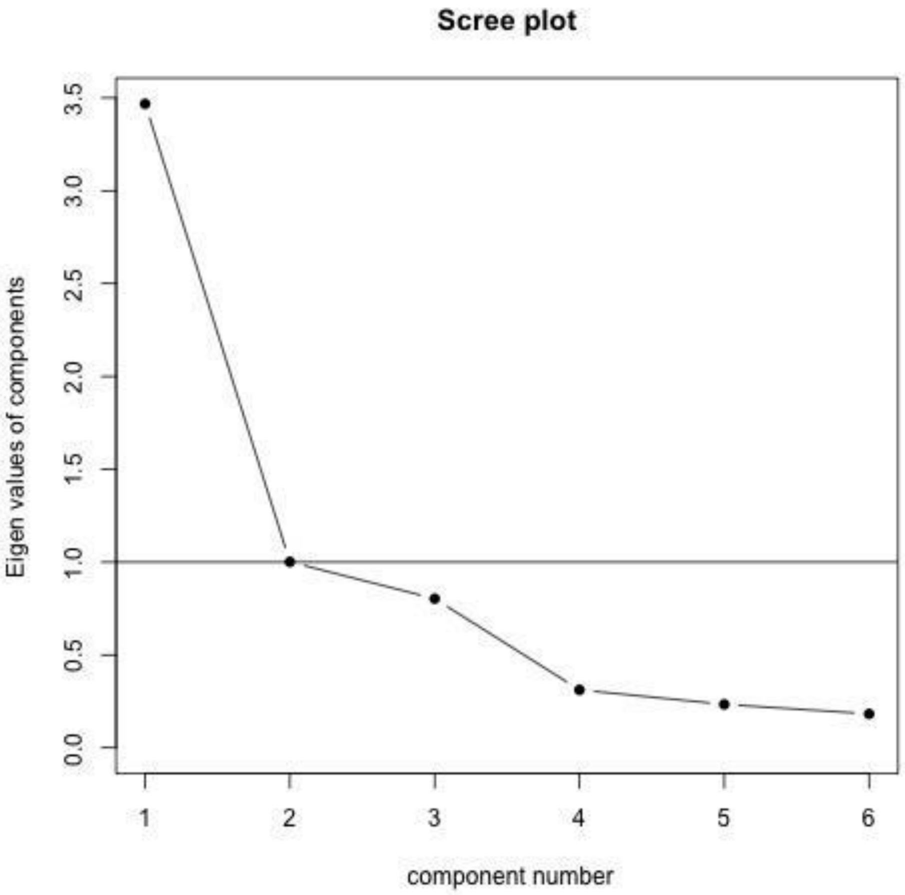

Fig S12. Scree plot of social data, showing two components with an eigenvalue greater than 1.

Table S16: Variable loadings of the social variables for a one and two principal component solution

	One PC	Two PC	
**Variable**	**PC1**	**PC1**	**PC2**
Subsistence	0.88	0.89	-0.02
Caste	0.55	0.55	0.05
Slavery	0.11	0.04	1.0
Class	0.90	0.90	0.10
Jurisdictional hierarchy	0.90	0.90	0.03
Community Size	0.87	0.88	-0.05

S1.9.5. Bivariate models

As described in the main text, for each bivariate relationship we test three models: a simple linear model; a model accounting for linguistic history; and a model accounting for spatial relationships.

Linguistic history is determined using a glottolog taxonomy [35]. As in [59], all language families are given a life of 6,000 years and language families are separated by a further 54,000 years, giving the tree a maximum time depth of 60,000 years. Branch lengths within language families are determined via Grafen branch length transformation. Linguistic covariance is accommodated into the regression using the following formula:

$$y = \beta_0 + \beta_1 \times x + e$$
$$e \sim N(0, V)$$

Using a Pagel's Lambda transformation, $V = \lambda V + (1 - \lambda)I$. Where V is a variance-covariance matrix derived from the phylogenetic tree.

Spatial relationships were modeled using mixed-effect models with spatial random effects. Locations are used at the society level (as opposed to song recording location) and are determined from the Global Jukebox metadata. We implement the following model:

$$y \sim N(\mu_i, \sigma^2)$$
$$\mu_i = X_i\beta + u(s_i)$$
$$u(s_i) \sim MVN(0, F(\Theta_1, \Theta_2, \ldots, \Theta_n))$$

F is defined by the Matérn correlation function, and is used to determine spatial covariance. Matérn parameters ν (rate of decay) and κ (smoothness) is estimated from the data. In all models ν was estimated to be 0.5, which approximates exponential decay in spatial covariance.

To compare the performance of each model variation, we use AIC comparison. We determine a difference of greater than -2 between models to indicate a statistically significant difference, a common rule of thumb. The AIC results are displayed in Table S17.

Table S17: Shows the N, Beta coefficients, and AIC values for each variation of the bivariate models.

		Beta coefficients			AIC		
**Correlation**	**N**	**Bi-Variate**	**Language**	**Geography**	**BV**	**LG**	**GEO**
Musical organization of the orchestra ~ Jurisdictional hierarchy	330	0.25***	0.16	0.17***	336.4	316.8	274.5
Text repetition ~ Subsistence	252	-0.47***	-0.40	-0.40***	110.7	112.3	98.4
Melodic Interval Size ~ Community Size	221	-0.20***	-0.12	-0.14*	-91.5	-107.7	-103.7
Embellishment (rev) ~ Class + Caste + Slavery	295	0.17***; 0.30***; -0.01	0.15*; 0.14; 0.01***	0.16***; 0.23***; 0.04	102.5	69.0	47.97
Enunciation (rev) ~ Jurisdictional hierarchy	330	0.23***	0.17***	0.16***	-22.5	-77.9	-79.6
Musical PC1 ~ Social PC1	147	0.64***	0.65***	0.60***	342.8	340.6	327.3

BV contains the results for a simple linear model, LG contains the results for a regression controlling for language phylogeny, GEO contains the results for a model controlling for spatial relationships.

Next we explore the model output from the best performing generalized linear mixed model for each bivariate model, and present a plot of the data. The plot shows the raw data, and a regression line determined by the coefficients of the best fitting model as determined by the lowest AIC in Table S17.

S1.9.6. Musical organization of the orchestra (CV7) vs Jurisdictional hierarchy

As expected, we find a significant positive relationship between the musical organization of the orchestra (CV7) and jurisdictional hierarchy. The best performing model also controlled for geographic regions ($\beta = 0.17$, $p < 0.001$) This suggests that as the number of levels in jurisdictional hierarchy increases, there is also an increase in the complexity of musical texture within the orchestra. (Fig S14).

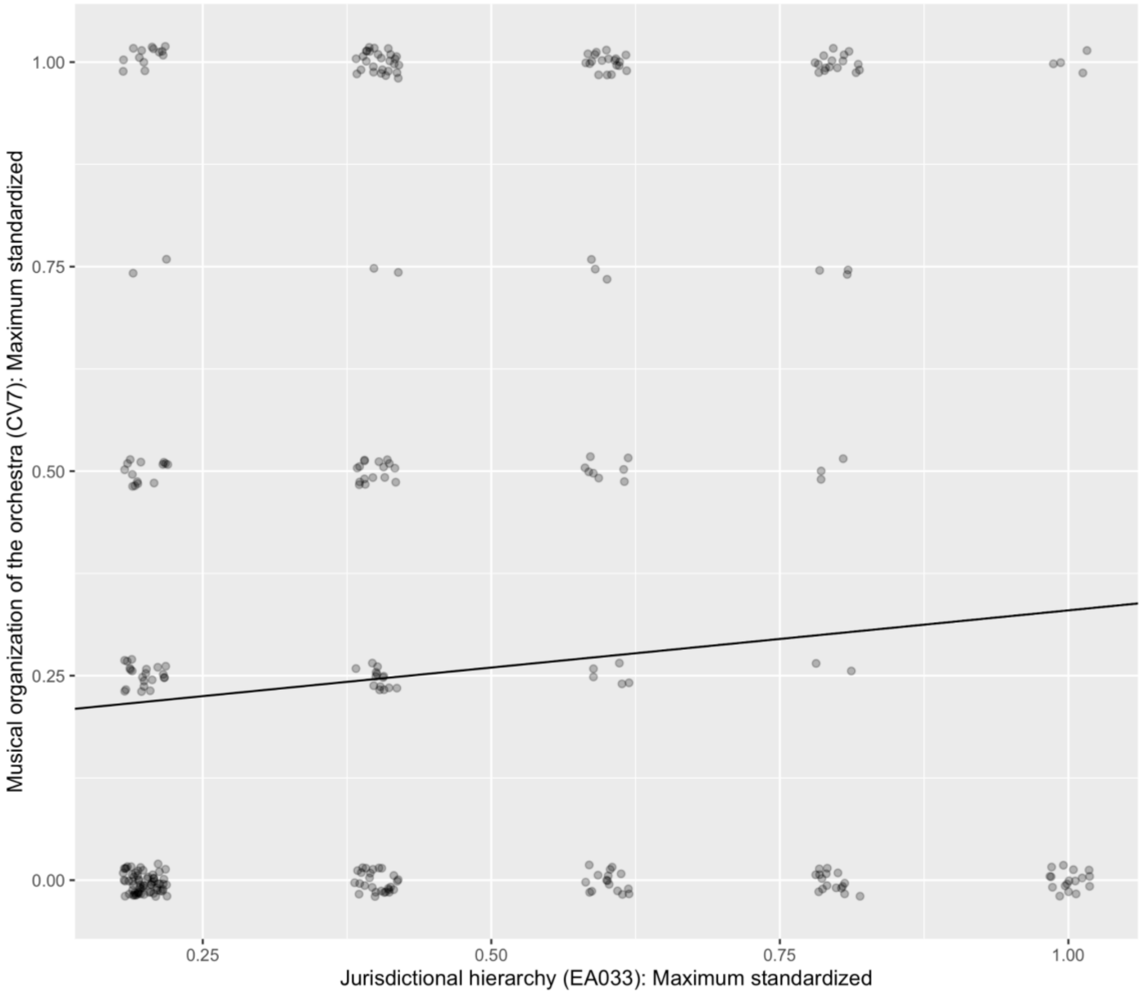

Fig S14. Scatter plot of the significant positive relationship between Musical organization of the orchestra (CV10) and Jurisdictional hierarchy.

S1.9.7 Repetition of Text (CV10) vs Subsistence

We find a significant negative relationship between CV8 and Subsistence types. A model that also controlled for geography explained the data best (β = -0.40, $p < 0.001$). This suggests that as a reliance on agriculture increases, we see less text repetition within a society, in line with Lomax' prediction (Fig S15).

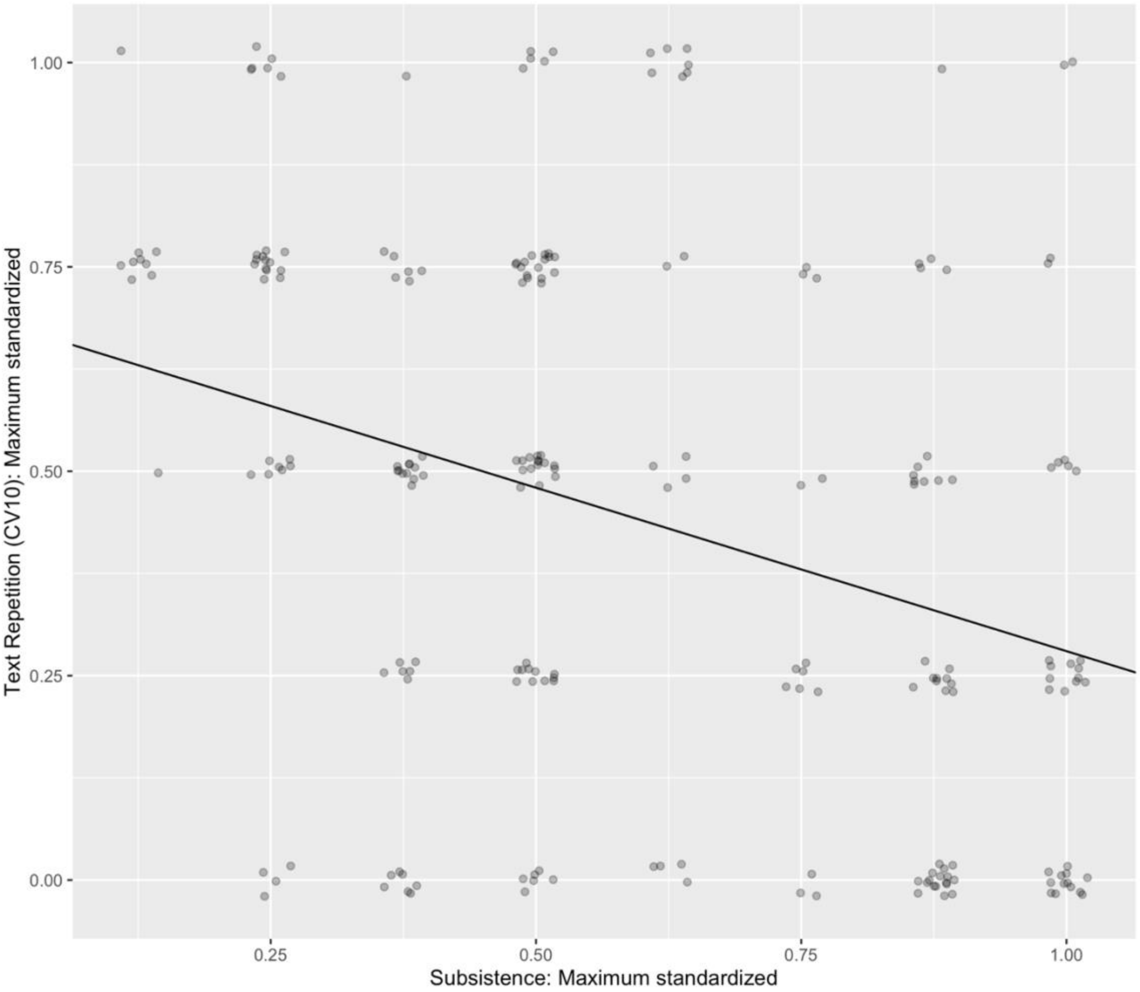

Fig S15. Scatter plot of the significant negative relationship between Text repetition (CV10) and Subsistence. Subsistence is an aggregated variable, whose calculation is described in Table S25.

S1.9.8. Interval size (CV21) vs community size (EA031)

We find a non-significant negative relationship between interval size and community size in the best fitting model, which controlled for language (β = -0.12, p = 0.011).

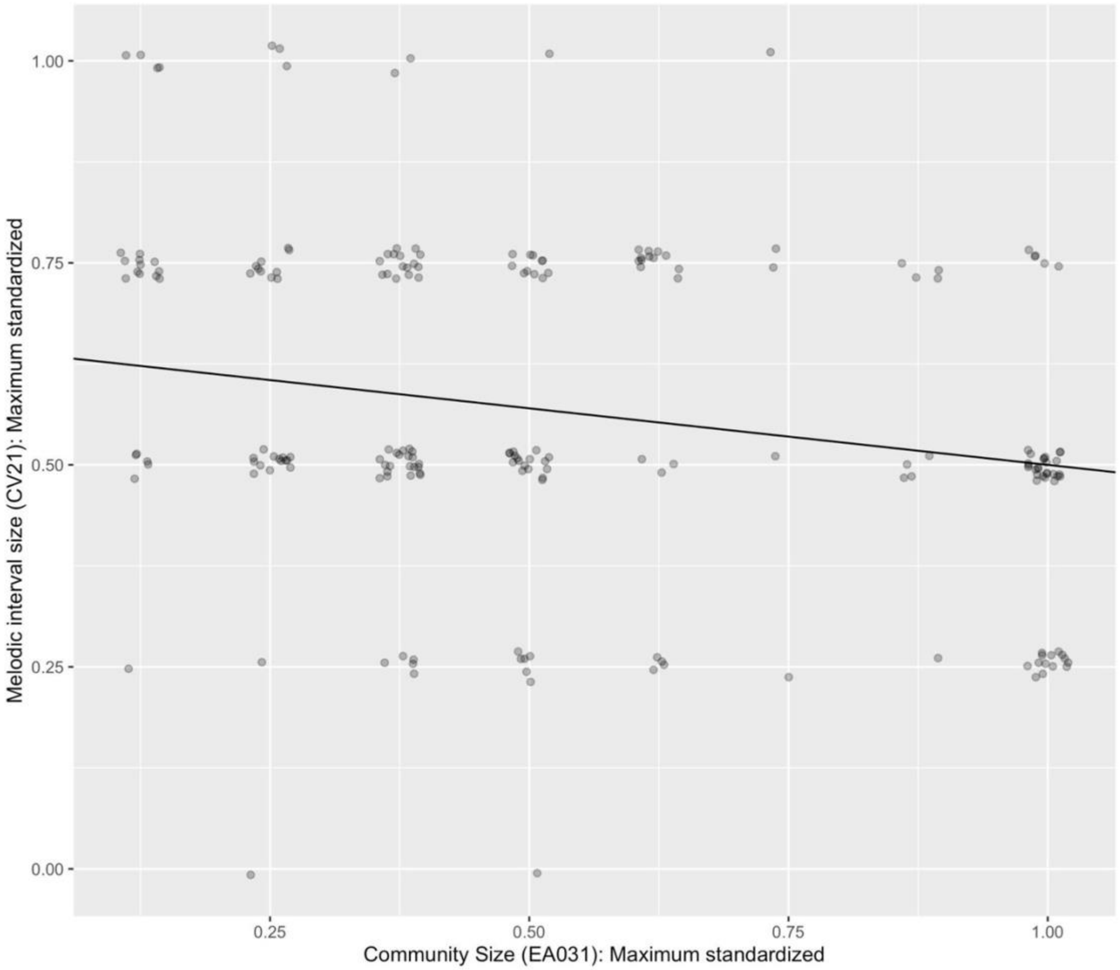

Fig S16. Scatterplot of the significant negative relationship between Melodic Interval size (CV21) against Community Size (EA031). Variable code definitions are described at the bottom of the document.

S1.9.9. Embellishment (CV23) vs class + caste + slavery

The next hypothesis we look at is between Embellishment and three measures of social hierarchy, also known as social layering. Previous correlations aggregated these variables, but a PCA of these three variables suggested these variables were largely orthogonal, meaning aggregating would be inappropriate. Since we have a multi-dimensional model, we present the data in a series of three graphs. Each graph shows a bivariate relationship between Embellishment and one of the three social variables. We then plot four regression lines showing the relative impact of the other two variables and different strengths of relationship (high levels or low levels). Note that Embellishment has been reverse coded. High values on the original scale indicate low embellishment,

now high values indicate high embellishment. In all cases a model that also controlled for geographic relationships predicted the data best.

As expected, we find a significant positive relationship between Embellishment and Class ($\beta = 0.16$, $p < 0.001$). This suggests that as a society has more levels than Class levels, there is more Embellishment. Lomax only explored this relationship on aggregate, so there is no direct comparison here, however, he had predicted that embellishment was reflective of stratification. We also see a significant influence of the Caste on the relationship between Class and Embellishment. Note that when there is a high level of Caste differentiation the regression line drops lower (red and blue line), compared to when there is less caste differentiation (green and purple). Changes in rates of slavery however, have little impact. These effects are reflected in the significant Caste effect and significant Slavery effect, which we discuss next.

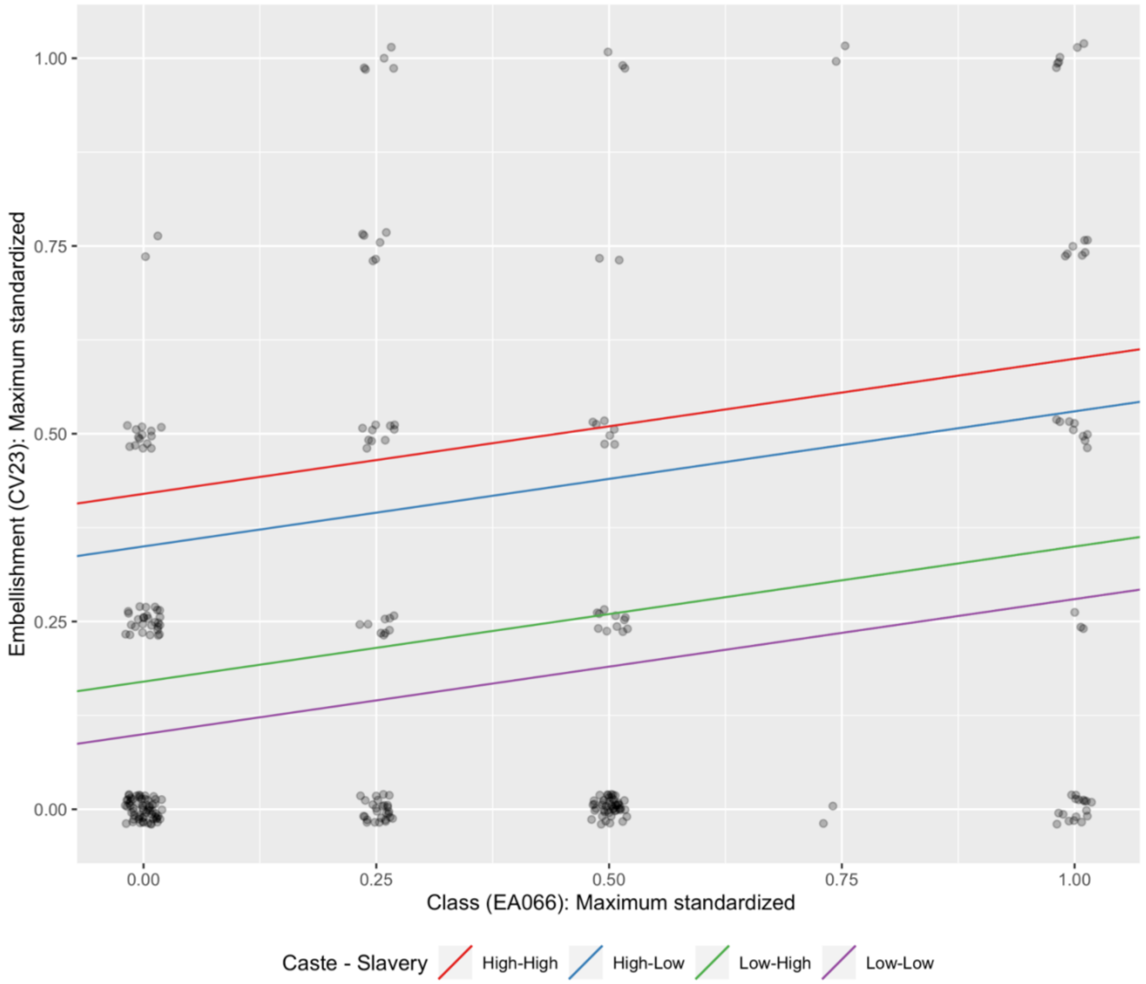

Fig S17. Scatterplot of the significant positive relationship between Embellishment (CV23) and Class (EA066). This model shows four regressions lines describing the additional effects of Caste and Slavery in the model. The lines show high and low combinations of each variable, detailed in the legend.

We find a significant positive relationship between Embellishment and Caste (Fig S18; β = 0.23, p < 0.001). This suggests that as the number of Caste distinctions increase, there is more Embellishment. We show lines representing the variability of the other two variables in the model. Here we see that changes in Class seem to influence the relationship to some degree, but to a lesser extent that Caste mediated the Class relationship. Notably, when we compare the strength of the correlation, Caste is approximately twice as strong as Class. Slavery shows a weaker, and non-significant relationship, as shown in Fig S19 (β = 0.04, p = 0.88).

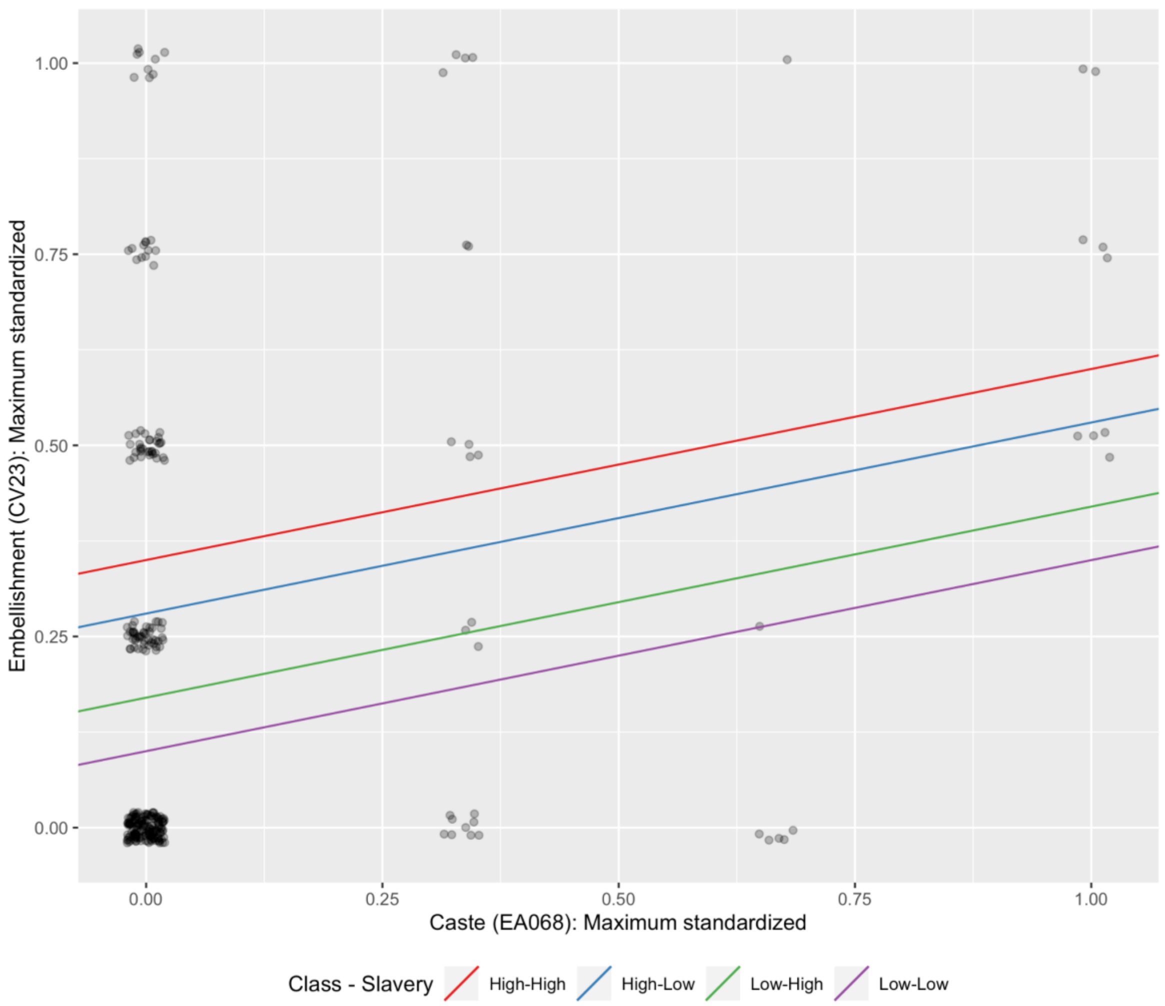

Fig S18. Scatterplot of the significant positive relationship between Embellishment (CV23) and Caste (EA068). This model shows four regression lines describing the additional effects of Class and Caste in the model. The lines show high and low combinations of each variable, detailed in the legend. The model contains random intercepts for geographical region, and the regression lines assume societies from East Africa.

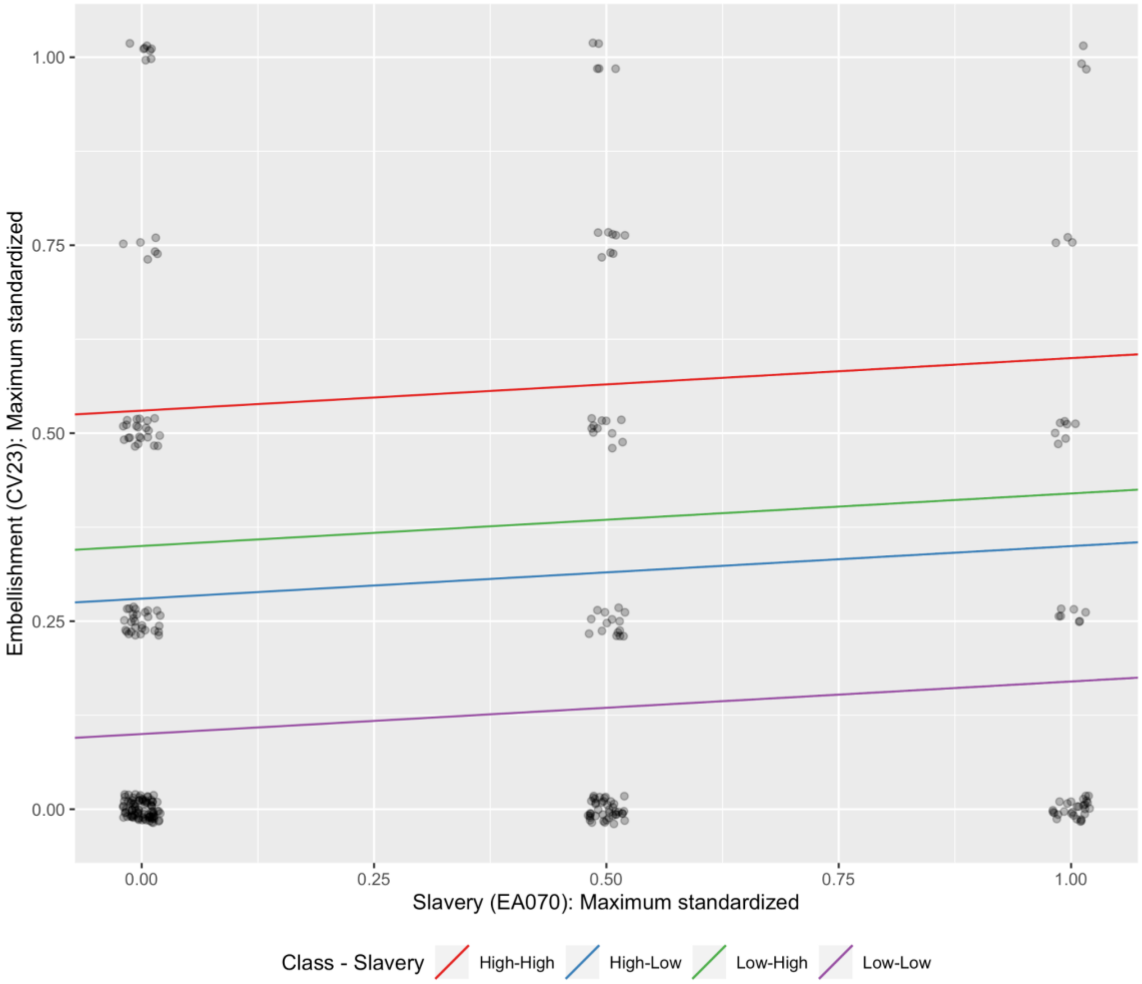

Fig S19. Scatterplot of the non-significant relationship between Embellishment (CV23) and Slavery (EA070). This model shows four regression lines describing additional effects of Class and Caste in the model. The lines show high and low combinations of each variable, detailed in the legend. The model contains random intercepts for geographical region, and the regression lines assume societies from East Africa.

S1.9.10. Enunciation (CV37) vs jurisdictional hierarchy

We find a significant positive relationship between reverse coded Enunciation and Jurisdictional hierarchy in a model also containing information on geographic relationships (β = 0.16, p < 0.001). This suggests that an increase in the number of hierarchies would show an increase in precision. of enunciation.

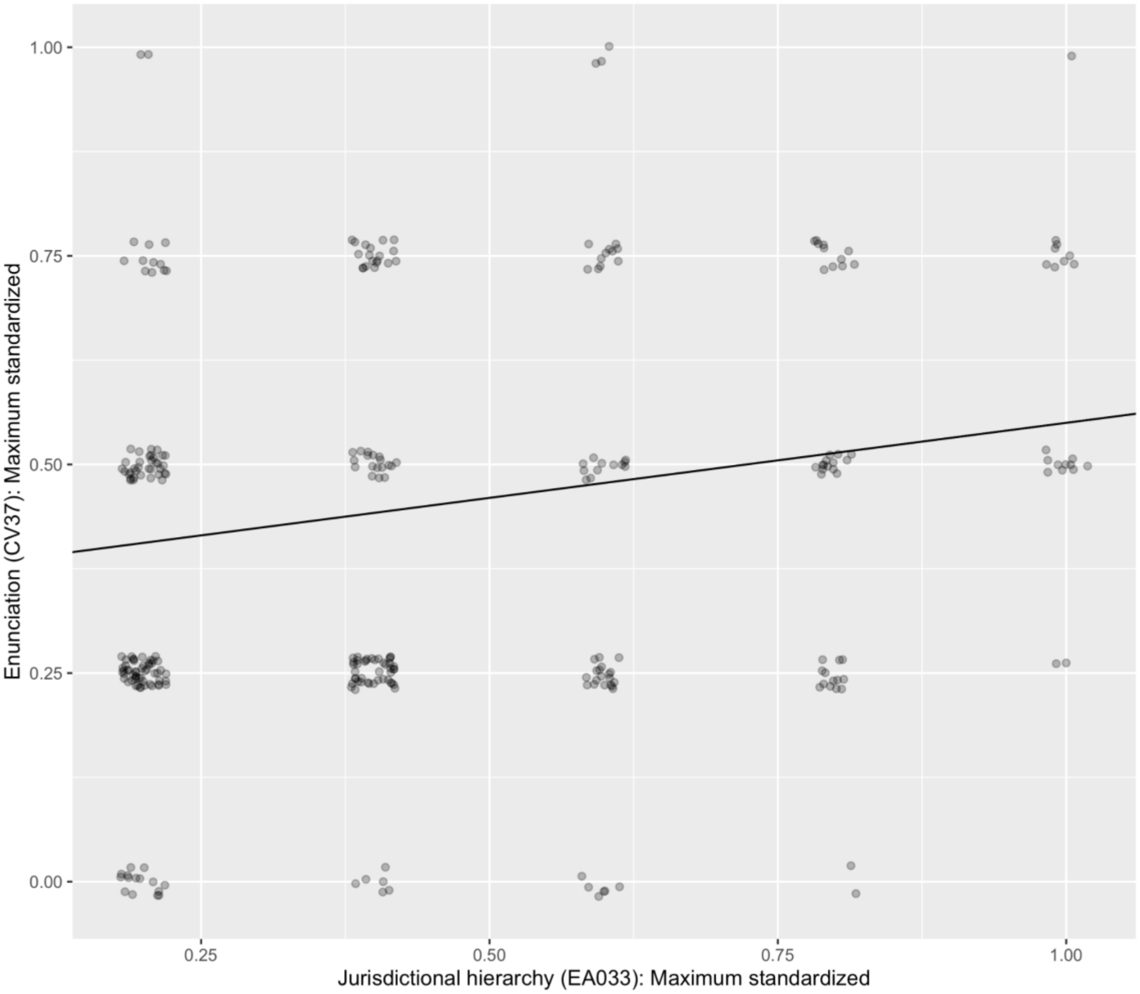

Fig S20. Scatterplot of the significant positive relationship between reverse coded Enunciation (CV23) and Jurisdictional hierarchy beyond the local community (EA033).

S1.9.11. Variable codes

Below are tables showing the original codes and descriptions for all variables used in the correlations. For Cantometric variables used in the correlation, we use the modal value across all songs within a society (described in S1.9.1). Both Cantometric and Ethnographic Atlas variables are then maximum standardized (i.e. divided by the largest value) before analysis. Embellishment and Enunciation are both reverse coded by subtracting their values from 1.

<u>**Musical Variables**</u>

Table S18. Musical organization of the Orchestra (CV7)

Code	Description
1	Non-occurrence. No instruments or Two or more instruments perceived to be totally asynchronous.
4	Monophony. One instrument playing one note at a time, or in octaves.
7	Unison. A group of instruments playing the same melody in unison or in octaves, an instrumental solo with simple percussion accompaniment—drum, rattle, sticks, clapping, etc., or any untuned percussion ensemble unless playing in polyrhythm, in which case, code 13 (Polyrhythm) (see Point 5).
10	Heterophony. Each instrument plays the same melody in a slightly different manner. The variation is usually rhythmic, with some instruments trailing behind, others pushing forward; or with some instruments more rhythmically active than others.
13	Polyphony or polyrhythm. The use of simultaneously produced intervals other than unison or the octave. Two-part intervals of this kind are considered polyphony, as well as harmonies of greater complexity.

Table S19. Text repetition (CV10)

Code	Description
1	Little or no repetition—wordy. A continuous stream of dissimilar sung syllables, words, and phrases, with little or no repetition or use of non-lexical utterances. In such songs—epics, ballads, songs of prayer and supplication, and much of Western and Eurasian song—text is of paramount importance.
4	Some repetition. Some repetition and/or the use of non-lexical utterances—about one fourth repeated text.
7	Half repetition. A substantial amount of repetition and/or non-lexical utterances that more or less equals the flow of unrepeated words.
10	Quite repetitious. Considerably more than half (about two-thirds) of the sung performance is accounted for by repetition and/or non-lexical utterances.
13	Extreme repetition. The text seems to be almost entirely composed of repetition of some kind and/or non-lexical utterances.

Table S20. Melodic Interval size (CV21)

Code	Description
1	Monotone. No intervals occur. The song remains on approximately one pitch. A polyphonic song would be coded “monotone” if each part stays at the same pitch level.

4	Narrow intervals. Intervals of a half step or less are prominent (though not necessarily predominant) in the song.
7	Diatonic intervals. Diatonic melodies where whole step predominates.
10	Large intervals. Intervals of a third occur more frequently than other intervals.
13	Very large intervals. Intervals of a fourth and a fifth or larger predominate.

Table S21. Embellishment (CV23)

**Original Code**	**Description**
**1**	Extreme embellishment.
**4**	Much embellishment.
**7**	Medium or considerable embellishment.
**10**	Slight embellishment.
**13**	Little or no embellishment.

Note: Embellishment was reverse coded for the correlation analyses, meaning smaller values equate to less embellishment. Shown above is the original code.

Table S22. Enunciation (CV37)

**Original Code**	**Description**
**1**	Very precise enunciation. Highly articulated consonants and syllables. This is generally typical of the storytelling singers of Eurasian polities.
**4**	Precise enunciation. Clearly articulated consonants in sung texts. Here one listens to the whole consonantal range and makes certain that all consonants are easily discernible.
**7**	Moderate enunciation. A moderate degree of enunciation.
**10**	Softened enunciation. Consonants are hard to distinguish and syllables are run together to some degree.
**13**	Very softened enunciation. Situations in which consonants are absent or nearly absent from the text, and/or in which syllables are run together.

Note: Enunciation was reverse coded for the correlation analyses. Shown above is the original code.

Social variables

Table S23. Jurisdictional hierarchy beyond local community (EA033)

Code	Description
**1**	No political authority beyond community (e.g., autonomous bands and villages)
**2**	One level (e.g., petty chiefdoms)
**3**	Two levels (e.g., larger chiefdoms)
**4**	Three levels (e.g., states)
**5**	Four levels (e.g., large states)

Table S24. Community Size (EA031)

Code	Description
**1**	Fewer than 50 persons
**2**	From 50 to 99 persons
**3**	From 100 to 199 persons
**4**	From 200 to 399 persons
**5**	From 400 to 1,000 persons
**6**	More than 1,000 persons in the absence of indigenous urban aggregations
**7**	One or more indigenous towns of more than 5,000 inhabitants but none of more than 50,000
**8**	One or more indigenous cities with more than 50,000 inhabitants

Table S25. Subsistence

Code	Description	Original Lomax Scale	Revised scale

**1**	Collecting outweighs game producing and agriculture	(EA004 < 4 and EA005 < 4 and EA004 + EA005 < 6 and the greatest value of (E001, E002, E003) > the greater value of (EA004, EA005)) and either (EA001 ≥ 4 and EA001 ≥ EA002 and EA003 - EA001 ≤ 1) or (EA001 < 4 and EA001 > EA002 > EA003 or EA001 > EA003 > EA002)	(EA004 < 4 and EA005 < 4 and EA004 + EA005 < 6 and the greatest value of (E001, E002, E003) > the greater value of (EA004, EA005)) and either (EA001 ≥ 4 and EA001 ≥ EA002 and EA003 - EA001 ≤ 1) or (EA001 < 4 and EA001 > EA002 > EA003 or EA001 > EA003 > EA002)
**2**	Hunting and/or fishing outweigh collection and/or agriculture	(EA004 < 4 and EA005 < 4 and EA004 + EA005 < 6 and the greatest value of (E001, E002, E003) > the greater value of (EA004, EA005)) and does not satisfy the conditions for 1	(EA004 < 4 and EA005 < 4 and EA004 + EA005 < 6 and the greatest value of (E001, E002, E003) > the greater value of (EA004, EA005)) and does not satisfy the conditions for 1
**3**	Planters (prior to European contact) with simple tools and no large domestic animals	Does not satisfy conditions for 1, 2, 3, 4, 5, 6, 7, or 8.	EA040 = 1-3 and EA005 > 4 and EA028 =3 or EA040-1-2 and EA005 > 4 and EA003 < 3 and EA028=4
**4**	Cultivators with simple tools and animal husbandry (goats, sheep, horses, deer, camels, yaks, water buffalo, or cattle)	(EA040 > 1 and EA005 > 0) and either (EA028 = 4, 5, or 6 and EA039 ≠ 3) or (EA028 = 3).	(EA040 > 2 and EA005 > 4) and either (EA028 = 5 and EA039 ≠ 3) or (EA028 = 3) or (EA028=4 and EA003<3)
**5**	Full nomadic pastoralism, at least 70% dependent on animal husbandry	EA004 > 5 and EA005 < 3	EA004 > 5 and EA005 < 3
**6**	Horticulture with fishing, tree cultivation, animal	EA040 > 1 and EA003 > 2 and EA028 = 4	EA040 > 1 and EA003 > 2 and EA028 = 4 and EA005 > 4

	husbandry, and ocean fishing		
**7**	Plough agriculture	EA028 = 5 and EA039 = 3 and EA040 = 3 or 7 and EA005 > (EA004)/2	EA028 = 5 and EA039 = 3 and EA040 = 3 or 7 and EA005 > 4
**8**	Irrigation	EA028 = 6 and EA039 = 3 and EA040 = 3 or 7	EA028 = 6 and EA039 = 3 and EA040 = 3 or 7 and EA005 > 4

Table S26. Class (EA066)

Code	Description
**1**	Absence of significant class distinctions among freemen (slavery is treated in EA070), ignoring variations in individual repute achieved through skill, valor, piety, or wisdom
**2**	Wealth distinctions, based on the possession or distribution of property, present and socially important but not crystallized into distinct and hereditary social classes
**3**	Elite stratification, in which an elite class derives its superior status from, and perpetuates it through, control over scarce resources, particularly land, and is thereby differentiated from a property-less proletariat or serf class
**4**	Dual stratification into a hereditary aristocracy and a lower class of ordinary commoners or freemen, where traditionally ascribed noble status is at least as decisive as control over scarce resources
**5**	Complex stratification into social classes correlated in large measure with extensive differentiation of occupational statuses

Table S27. Caste (EA068)

Code	Description
**1**	Caste distinctions absent or insignificant
**2**	One or more despised occupational groups, e.g., smiths or leather workers, distinguished from the general population, regarded as outcastes by the latter, and characterized by strict endogamy

**3**	Ethnic stratification, in which a superordinate caste withholds privileges from and refuses to intermarry with a subordinate caste (or castes) which it stigmatizes as ethnically alien, e.g., as descended from a conquered and culturally inferior indigenous population, from former slaves, or from foreign immigrants of different race and/or culture
**4**	Complex caste stratification in which occupational differentiation emphasizes hereditary ascription and endogamy to the near exclusion of achievable class statuses

Table S28. **Slavery (EA070)**

Code	Description
**1**	Absence or near absence of slavery
**2**	Incipient or nonhereditary slavery, i.e., where slave status is temporary and not transmitted to the children of slaves
**3**	Slavery reported but not identified as hereditary or nonhereditary
**4**	Hereditary slavery present and of at least modest social significance

S1.10. The Global Jukebox and cultural equity

The Global Jukebox advances cultural equity and social justice by sharing its resources with researchers and culture bearers alike, and by evaluating them not by Western standards, but according to criteria used variably by singers and musicians worldwide. It is a living repository of the world’s musical cultures, a platform for all forms of musical expression, and a source of inspiration for young musicians. The musical traditions it contains are endangered or no longer practiced. The Global Jukebox validates and sustains these historical musical practices and keeps their memory present. It is always expanding as new items of music and dance are added and eventually coded, new apps and educational offerings, new Journeys, new analyses are visualized, and new ways of looking at the data are added. Assuring its longevity is an interactive world music course using Cantometrics and based on deep listening, that trains researchers to code, or sensitizes students and casual users to the aesthetic preferences of the world’s peoples, and familiarizes them with the myriad ways the “musical voice” is handled throughout the world.

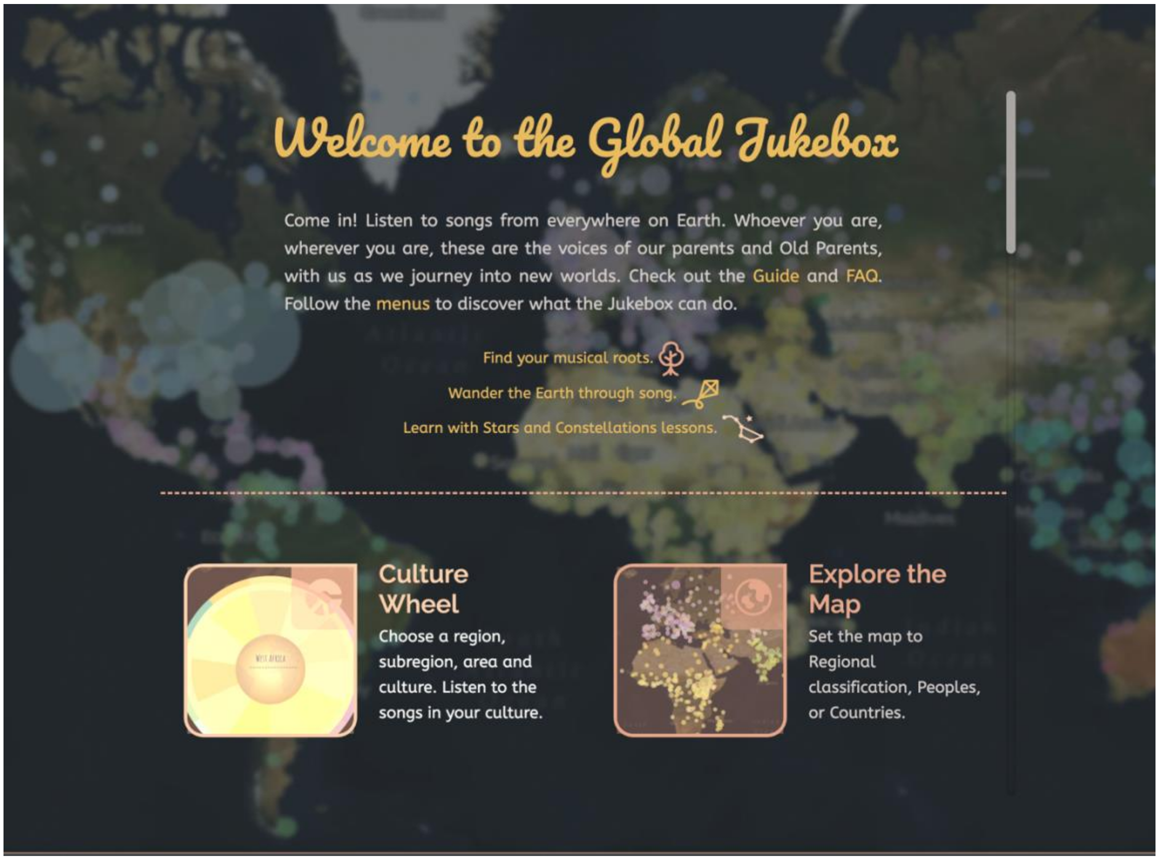

Fig S21. Welcome screen of the Global Jukebox.

S1.11. References

1. Wood, A. L. C. (2018a). "Like a cry from the heart": An insider's view of the genesis of Alan Lomax's ideas and the legacy of his research, Part I. Ethnomusicology, 62(2), 230-264.

2. Arensberg, C. M. (1972). Culture as behavior: Structure, and emergence. Annual Review of Anthropology 1:1–27.

3. Birdwhistell, R. L. (1970). Kinesics and Context. Philadelphia: University of Pennsylvania Press.

4. Lomax, A., & Berkowitz, N. (1972). The evolutionary taxonomy of culture. Science, 177(4045), 228–239. https://doi.org/10.1126/science.177.4045.228

5. Savage, P. E. (2019). Cultural evolution of music. Palgrave Communications, 5(16), 1–12. https://doi.org/10.1057/s41599-019-0221-1

6. Savage, P. E., & Brown, S. (2013). Toward a new comparative musicology. *Analytical Approaches to World Music*, *2*(2), 148–197. https://doi.org/10.31234/osf.io/q3egp

7. Jordania, J. & Brown, S. (2013). Universals in the world's musics. Psychology of Music, 41(2), 229–48.

8. Savage, P.E. (2019). Universals. In J. L. Sturman (Ed.), The SAGE International Encyclopedia of Music and Culture (p. 2282-2285). SAGE Publications. http://doi.org/10.4135/9781483317731.n759

9. Mehr, S. A., Singh, M., Knox, D., Ketter, D. M., Pickens-Jones, D., Atwood, S., Lucas, C., Jacoby, N., Egner, A. A., Hopkins, E. J., Howard, R. M., Hartshorne, J. K., Jennings, M. V., Simson, J., Bainbridge, C. M., Pinker, S., O'Donnell, T. J., Krasnow, M. M., & Glowacki, L. (2019). Universality and diversity in human song. *Science, 366*, eaax0868. https://doi.org/10.1126/science.aax0868

10. Kristiansen, Kristian. 2014. Towards a new paradigm? The third science revolution and its possible consequences in archaeology. *Current Swedish Archeology*, vol. 22. Gothenburg.

11. Gray R. D., Watts J. (2017). Cultural macroevolution matters. Proceedings of the National Academy of Science, U. S. A. Jul 25;114(30):7846-7852. doi: 10.1073/pnas.1620746114.

12. Watts J, et al. (2015). Broad supernatural punishment but not moralizing high gods precede the evolution of political complexity in Austronesia. *Proc R Soc B Biol Sci* 282:20142556.

13. Watts J, Sheehan O, Atkinson Q. D., Bulbulia J, Gray R. D. (2016). Ritual human sacrifice promoted and sustained the evolution of stratified societies. *Nature* 532:228–231.

14. Passmore, S., Wood, A. L. C., Barbieri, C., Shilton, D., Daikoku, H., Atkinson, Q. D., & Savage, P. E. (2022). Global relationships between musical, linguistic, and genetic diversity. *PsyArXiv* preprint: https://doi.org/10.31234/osf.io/mdrsn

15. Lomax, A. (1980). Factors of musical style. In S. Diamond (Ed.), Theory & practice: Essays presented to Gene Weltfish (pp. 29–58). The Hague, Netherlands: Mouton.

16. Kubik, G. (2009). Africa and the Blues. Univ. Press of Mississippi.

17. Lomax, A. (1976). Cantometrics: An approach to the anthropology of music. Berkeley, CA: University of California Exten- sion Media Center.

18. Savage, P. E. (2018). Alan Lomax’s Cantometrics Project: A comprehensive review. Music & Science, 1, 1–19. https://doi.org/10.1177/2059204318786084

19. Ayres, B., E. Eriksen, C.M. Arensberg, A. Lomax. (c.1968). Social Factors Guide and Codebook, edited and updated by S. Silbert. Association for Cultural Equity. https://github.com/theglobaljukebox/socialfactors/tree/main/raw

20. Lomax, A. (1968). Folk song style and culture. Washington, DC: American Association for the Advancement of Science.

21. Reed, F. et al. (2009). A Comparison of genetic and musical affiliations in Sub Saharan africa. Paper given at the American Anthropological Association Meetings, Washington., D.C.

22. Reich, D. (2018). Who We Are and How We Got Here: Ancient DNA and the New Science of the Human Past. Pantheon Books, New York; Oxford University Press, Oxford.

23. Cavalli-Sforza, L. L., Menozzi, P., & Piazza, A. (1994). *The history and geography of human genes*. Princeton University Press

24. Cavalli-Sforza, L. L., & Feldman, M. W. (2003). The application of molecular genetic approaches to the study of human evolution. *Nature Genetics*, *33 Suppl*(March), 266–275. https://doi.org/10.1038/ng1113

25. Leroi, A. M., Mauch, M., Savage, P. E., Benetos, E., Bello, J., Panteli, M., Six, J., & Weyde, T. (2015). The deep history of music project. *Proceedings of the 5th International Workshop on Folk Music Analysis (FMA-15)*, 83–84. http://researchgate.net/publication/286926649_The_Deep_History_of_Music_Project

26. Flory, M. 2017. Cultures Clustered by Song Style. In Symposium on the Global Jukebox. 62nd Annual Meeting of the Society for Ethnomusicology, Denver. Accessible at www.theglobaljukebox.org.

27. Bahrami-rad, D., Becker, A., & Henrich, J. (2021). Tabulated nonsense? Testing the validity of the Ethnographic Atlas. *Economics Letters* 204. https://doi.org/10.1016/j.econlet.2021.109880]

28. Ember, Carol R., and Melvin Ember. "Climate, econiche, and sexuality: Influences on sonority in language." *American Anthropologist* 109, no. 1 (2007): 180-185.

29. Flory, M. J. (2017). Correlations Tests. Performance Style and Culture Collection 2, Alan Lomax Archive. Association for Cultural Equity, Hunter College, New York City.

30. Alesina, A., Giuliano, P., & Nunn, N. (2013). On the Origins of Gender Roles: Women and the Plough. *The Quarterly Journal of Economics*, *128*(2), 469–530. https://doi.org/10.1093/qje/qjt005

31. Ayres, B. (1973). Effects of Infant Carrying Practices on Rhythm in Music. Ethos, 1(4), 387-404. Retrieved April 23, 2021, from http://www.jstor.org/stable/640187

32. Laban, Rudolph, Lisa Ullman. (2011 [1950]). The Mastery of Movement. Pre Textos ed; 4th edition.

33. Lomax, A., Arensberg, C., Berleant-Schiller, R., Dole, G., Hippler, A., Jensen, K., . . . Turyahikayo-Rugyema, B. (1977). A Worldwide Evolutionary Classification of Cultures by Subsistence Systems [and Comments and Reply]. *Current Anthropology*, 18(4), 659-708. Retrieved April 15, 2021, from http://www.jstor.org/stable/2741506

34. Peel, M.C., Finlayson, B.L. & McMahon, T.A. (2007). Updated world map of the Köppen-Geiger climate classification. Hydrol. Earth Syst. Sci., 11, 1633-1644.

35. Hammarström, H. & Forkel, R. & Haspelmath, M. & Bank, S. (2021). *Glottolog 4.4*. Leipzig: Max Planck Institute for Evolutionary Anthropology. https://doi.org/10.5281/zenodo.4761960. (Available online at http://glottolog.org, Accessed on 2021-08-18.

36. Kirby, K. R., Gray, R. D., Greenhill, S. J., Jordan, F. M., Gomes-Ng, S., Bibiko, H.-J., Blasi, D. E., Botero, C. A., Bowern, C., Ember, C. R., Leehr, D., Low, B. S., McCarter, J., Divale, W., & Gavin, M. C. (2016). D-PLACE: A Global Database of Cultural, Linguistic and Environmental Diversity. Plos One, 11(7), e0158391. https://doi.org/10.1371/journal.pone.0158391

37. HRAF. *eHRAF World Cultures*. https://ehrafworldcultures.yale.edu/ehrafe/

38. Murdock, G.P. (1968). World Sampling Provinces. Ethnology, 7(3), 305-326. doi:10.2307/3772896

39. Passmore, S. (2020). The problem with Galton's Problem. https://www.sampassmore.com/post/galtons-problem/

40. Eff, E. A. (2004). Does Mr. Galton still have a problem? Autocorrelation in the Standard Cross-Cultural Sample. *World Cultures,* 15(2), 153–170.

41. Chairetakis, A. L. (1991). The past in the present: Community variation and earthquake recovery in the Sele Valley, Southern Italy, 1980-1989, Ph.D. Dissertation. Columbia University.

42. Del Rio, M. (1995). Programming guide for the GJB research programs. Providing information on the project history, database formats, the current software framework, and the programming techniques used. Association for Cultural Equity.

43. Lomax, A. (1989). Cantometrics. In International encyclopedia of communications (1st ed., pp. 230–233). New York, NY: Oxford University Press.

44. Rzeszutek, T., Savage, P. E., & Brown, S. (2012). The structure of cross-cultural musical diversity. Proceedings of the Royal Society B: Biological Sciences, 279(1733), 1606–1612. https://doi.org/10.1098/rspb.2011.1750

45. Brown, S., Savage, P. E., Ko, A. M.-S., Stoneking, M., Ko, Y.-C., Loo, J.-H., & Trejaut, J. A. (2014). Correlations in the population structure of music, genes and language. Proceedings of the Royal Society B: Biological Sciences, 281(1774), 20132072. https://doi.org/10.1098/rspb.2013.2072

46. Savage, P. E., & Brown, S. (2014). Mapping music: Cluster analysis of song-type frequencies within and between cultures. Ethnomusicology, 58(1), 133–155. https://doi.org/10.5406/ethnomusicology.58.1.0133

47. Forkel, R., List, J.-M., Greenhill, S. J., Rzymski, C., Bank, S., Cysouw, M., … Gray, R. D. (2018). Cross-Linguistic Data Formats, advancing data sharing and re-use in comparative linguistics. Scientific Data, 5(1), 1–10. https://doi.org/10.1038/sdata.2018.205

48. Nettl, B. (1970). Review of A. Lomax, Folk song style and culture. *American Anthropologist*, 72(2), 438–441.

49. Wood, A. L. C. (2018b). “Like a cry from the heart”: An insider’s view on the genesis of Alan Lomax’s ideas and the legacy of his research: Part II. Ethnomusicology, 62(3), 403-438.

50. Daikoku, H., Shenghao, D., Sanne, U. S., Benetos, E., Wood, A. L. C., Shimozono, T., Fujii, S., & Savage, P. E. (2022). Human and automated estimates of musical similarity in a global sample. In *Proceedings of the 10th International Workshop on Folk Music Analysis (FMA2022)*, 26-32. http://doi.org/10.5281/zenodo.7100288

51. Savage, P. E., Merritt, E., Rzeszutek, T., & Brown, S. (2012). CantoCore: A new cross-cultural song classification scheme. *Analytical Approaches to World Music*, *2*(1), 87–137. https://doi.org/10.31234/osf.io/s9ryg

52. Savage, P. E., Brown, S., Sakai, E., & Currie, T. E. (2015). Statistical universals reveal the structures and functions of human music. *Proceedings of the National Academy of Sciences of the United States of America*, *112*(29), 8987–8992. https://doi.org/10.1073/pnas.1414495112

53. Landis, J. R., & Koch, G. G. (1977). The measurement of observer agreement for categorical data. *Biometrics*, *33*(1), 159–174.

54. Sim, J., & Wright, C. C. (2005). The kappa statistic in reliability studies: Use, interpretation, and sample size requirements. *Physical Therapy*, *85*(3), 257–268.

55. McHugh, M. L. (2012). Interrater reliability: The kappa statistic. *Biochemica Medica*, *22*(3), 276–282.

56. M. Marolt, C. Bohak, A. Kavčič, and M. Pesek Automatic segmentation of ethnomusicological field recordings. *Applied sciences*, vol. 9, iss. 3, pp. 1-12, 2019, https://doi.org/10.3390/app9030439

57. Mauch, M. and Dixon, S., 2014, May. pYIN: A fundamental frequency estimator using probabilistic threshold distributions. In *2014 IEEE International*

Conference on Acoustics, Speech and Signal Processing (ICASSP) (pp. 659-663). IEEE.

58. Murdock, G.P. Ethnographic Atlas, Installments I-XXVII. Ethnology. 1–10.

59. Roberts, S. G., Winters, J., & Chen, K. (2015). Future Tense and Economic Decisions: Controlling for Cultural Evolution. PLOS ONE, 10(7), e0132145. https://doi.org/10.1371/journal.pone.0132145

60. McDermott, J. H., Schultz, A. F., Undurraga, E. A., & Godoy, R. A. (2016). Indifference to dissonance in native Amazonians reveals cultural variation in music perception. *Nature*, *535*, 547–550. https://doi.org/10.1038/nature18635
